# Pan-cancer multi-omics analysis of PTBP1 reveals it as an inflammatory, progressive and prognostic marker in glioma

**DOI:** 10.1038/s41598-024-64979-5

**Published:** 2024-06-25

**Authors:** Zheng Ye, Yan Zhong, Zhiyuan Zhang

**Affiliations:** 1 Institute of Computational Science and Technology, Guangzhou University, Guangzhou, 510006 Guangdong China; 2 People’s Hospital of Dongxihu District, Wuhan, China; 3 Department of Neurosurgery, Jinling Hospital, Affiliated Hospital of Medical School, Nanjing University, Nanjing, China; 4 Zhongda Hospital, Southeast University, Nanjing, China

**Keywords:** Pan-cancer, PTBP1, Low grade glioma, Prognosis, Biomarker, Cancer genomics, Cancer microenvironment, Tumour biomarkers

## Abstract

PTBP1 is an oncogene that regulates the splicing of precursor mRNA. However, the relationship between PTBP1 expression and gene methylation, cancer prognosis, and tumor microenvironment remains unclear. The expression profiles of PTBP1 across various cancers were derived from the TCGA, as well as the GTEx and CGGA databases. The CGGA mRNA_325, CGGA mRNA_301, and CGGA mRNA_693 datasets were utilized as validation cohorts. Immune cell infiltration scores were approximated using the TIMER 2.0 tool. Functional enrichment analysis for groups with high and low PTBP1 expression was conducted using Gene Set Enrichment Analysis (GSEA). Methylation data were predominantly sourced from the SMART and Mexpress databases. Linked-omics analysis was employed to perform functional enrichment analysis of genes related to PTBP1 methylation, as well as to conduct protein functional enrichment analysis. Single-cell transcriptome analysis and spatial transcriptome analysis were carried out using Seurat version 4.10. Compared to normal tissues, PTBP1 is significantly overexpressed and hypomethylated in various cancers. It is implicated in prognosis, immune cell infiltration, immune checkpoint expression, genomic variation, tumor neoantigen load, and tumor mutational burden across a spectrum of cancers, with particularly notable effects in low-grade gliomas. In the context of gliomas, PTBP1 expression correlates with WHO grade and IDH1 mutation status. PTBP1 expression and methylation play an important role in a variety of cancers. PTBP1 can be used as a marker of inflammation, progression and prognosis in gliomas.

## Introduction

PTBP1 is a subfamily of ubiquitously expressed heterogeneous ribonucleoproteins (hnRNPs) located on chromosome 19. The protein encoded by this gene plays a key role in the pre-mRNA splicing process^1^. It is well known that abnormal alternative splicing can lead to the development of cancer^2^. Besides affecting the splicing of pre-mRNAs, PTBP1 is also involved in transport and metabolism of mRNA^3^. PTBP1 can participate in many aspects of cell metabolism, such as the growth and differentiation of neuronal cells, T cell activation, spermatogenesis, embryonic development. Among these biological processes, PTBP1 plays the most crucial role in the growth and differentiation of neuronal cells. In cancer cells, PTBP1 is mainly involved in the process of glycolysis^4^. The occurrence, apoptosis, proliferation, migration, and invasion of cancer are all regulated by PTBP1^5^.

Brain Lower Grade Glioma (LGG) is a highly invasive tumor that develops from healthy glial cells in the brain^6^. Due to its high degree of invasiveness, it is almost impossible to remove it through complete neurosurgery^7^. The remaining small lesions can still cause the recurrence and malignant progression of LGG. After LGG diffuses locally, its tumor cells exhibit a high degree of heterogeneity^8^. Some cells can develop into malignant glioma within a few months, threatening the health of patients, while other glioma cells can remain stable for several years. The heterogeneity of LGG cancer cells leads to greater variation in patient survival^9–11^. Therefore, clinically, genetic classification is increasingly relied upon to guide clinical treatment. For example, IDH^12^, TP53^13,14^, ATRX^15^ mutations are the molecular characteristics of most LGG patients and are also vital indicators of clinical behavior. Nevertheless, the diagnostic effectiveness of these mutations still needs more clinical data to support. Clinically, more molecular markers are still needed to assist clinical treatment decisions.

In this study, based on multi-omics analysis of the TCGA and CGGA databases, we clarified the relationship between PTBP1 mRNA expression and gene methylation, tumor immune microenvironment, tumor neo-antigen burden, and tumor mutation in various types of cancer. By focusing on low-grade gliomas, the study found that PTBP1 could be used as a molecular marker of LGG to indicate cancer progression, immune infiltration, and prognosis.

## Methods

### The expression profile and methylation profile of PTBP1 mRNA expression in pan-cancer

The UCSC Xena (https://xenabrowser.net) database^16^ was used to download the expression profile data of TCGA^17^ and GTEx^18^. Since there are limited number of normal samples in TCGA, we integrated the data of normal tissues from the GTEx database and the data of TCGA tumor tissues to analyze the expression differences of 27 tumors. The SMART App^19^ (http://www.bioinfo-zs.com/smartapp/) was used to download the beta values of PTBP1 methylation sites in 33 cancers. For each type of cancer, we calculated the differences in PTBP1 expression and methylation levels between normal and tumor tissues using the Wilcoxon test. The the LinkedOmics database was utilized to investigate genes associated with PTBP1 methylation. Specifically, we identified methylation genes correlated with PTBP1 gene expression in the TCGA LGG/GBM cohort through Spearman correlation analysis. Webgestalt, the WEB-based GEne SeT AnaLysis Toolkit, was employed for gene set enrichment analysis of these methylation-associated genes. During the calculation process, genes were ranked based on the Spearman correlation coefficient, followed by functional enrichment analysis using Gene Set Enrichment Analysis (GSEA).

### Tumor mutation burden, neo-antigen burden data analysis

TCGA tumor mutation burden data and neo-antigen burden data for all 33 cancers are available at https://gdc.cancer.gov/about-data/publications/panimmune^20^. The publication page (https://gdc.cancer.gov/about-data/publications/pancanatlas) provides access to the standardized, normalized, batch-corrected, and platform-corrected data matrices and mutation data generated by the PanCancer Atlas consortium. The Spearman correlation between PTBP1 expression and tumor mutation burden, neo-antigen burden was calculated for each of the 33 cancers. The R package “mfsb” was used for radar plotting.

### Drug sensitivity analysis

To explore the correlation between PTBP1 at the pan-cancer level and drug sensitivity, We collected the IC50 of 265 small molecules in 860 cell lines, and its corresponding mRNA gene expression from Genomics of Drug Sensitivity in Cancer (GDSC)^21^. The mRNA expression data and drug sensitivity data were merged. Pearson correlation analysis was performed to get the correlation between gene mRNA expression and drug IC50. The p-values were adjusted using the false discovery rate (FDR). We also performed the same analysis in the Genomics of Therapeutics Response Portal^22^ (CTRP, IC50 of 481 small molecules in 1001 cell lines) dataset. We screened for drugs with FDR < 0.01 for visualization.

### Immune score

According to the expression profile data of TCGA, the immune infiltration was calculated using MCPcounter and ESTIMATE^23,^^24^, respectively. The TIMER^25^ database was used to get the infiltration of six immune cells, including B cells, CD4 + T cells, CD8 + T cells, neutrophils, macrophages, and dendritic cells. The Cibersort algorithm was applied to assess the degree of infiltration of 22 lymphocyte types in the TCGA dataset^26^. The Pearson correlation coefficient between these immune cell infiltration scores and PTBP1 expression was first calculated and then correlation heat maps were plotted for each of the 33 cancers using the R package ggcorplot.

### Functional enrichment analysis

Gene Set Enrichment Analysis (GSEA)^27^ algorithm is used to perform functional enrichment analysis. The basic idea of GSEA is to use a predefined gene set (usually from functional annotations or the results of previous experiments) to rank the genes according to the degree of differential expression in the two types of samples and then check whether the preset gene set is in this ranking table Enrichment at the top or bottom. GSEA detects gene sets instead of individual gene expression changes so that these subtle expression changes can be included, and more ideal results are expected. We first divided PTBP1 into high and low PTBP1 expression groups based on the average expression of PTBP1 and used DESeq2 to calculate the fold change in gene expression between the two groups. Two gene sets, GO: BP^28^, and HALLMARK^29^, were obtained from the MSIGDB database^30^ and GSEA was performed based on differential expression foldchange between the two groups. Additionally, we used the LinkedOmics^31^ database to perform the GSEA algorithm based on genes correlated with the degree of PTBP1 methylation in TCGA-LGG cohort.

### Immunohistochemistry

Pathological sections included two patients each with Grade 2, Grade 3 and Grade 4 glioma stages, and three pathological sections were taken from each patient for immunohistochemical staining. The studies involving human participants were reviewed and approved by IEC for Clinical Research of Zhongda Hospital, Affilliated to Southeast University. The patients provided written informed consent to participate in this study. The monoclonal antibody mouse anti-human PTBP1 was purchased from Abway (1:200 dilution) and the chromogenic kit was purchased from DAKO (K5007, 1:100 dilution). PBS buffer (Na_2_HPO_4_ 8.1 mmol/L, KH_2_PO_4_ 4.5 mmol/L, NaCl 137 mmol/L, KCl 2.7 mmol/L, pH 7.4) was shaken and washed. The sections were then sealed by dehydration in the conventional way.

### Single-cell transcriptome and spatial transcriptome data processing pipeline

To assess the cytological profile of PTBP1 at the single cell level, we obtained expression profile data from 4058 glioma cells in the GSE102130 dataset^32^. Seurat version 4.10^33^ was used to analyze single-cell sequencing data from gliomas. In this dataset, there are five main cell types in glioma tissue, namely AC-like Malignant, Mono/Macro, OC-like Malignant, Oligodendrocyte, and OPC-like Malignant. To further analyze PTBP1 expression at the spatial transcriptome level, we obtained spatial transcriptome data for gliomas from the 10xGenomics official website. The data were clustered into groups using the Seurat spatial transcriptome analysis process and the cells of the spatial transcriptome were annotated using the annotation information from GSE102130.

### Statistical method

The survival curve and ROC curve were drawn by survminer^34^ and pROC^35^ software package using the R package. ESTIMATE and MCPcounter based on the R package are used to calculate the immune score of the genome. The R package ggpubr was used to plot box plots and perform statistical tests. the R package regplot was used to plot Nomograms. The Pearson correlation coefficient calculates the correlation of gene expression, and the correlation between tumor mutation load, tumor neo-antigen antigen, methylation site, and gene expression uses the Spearman correlation coefficient. P < 0.05 was considered statistically significant (*P < 0.05, **P < 0.01, ***P < 0.001, ****P < 0.0001, ns Not Significant). The workflow for this research is shown in Fig. S12.

## Results

### PTBP1 is highly expressed and hypermethylated in a variety of cancers

**Figure 1:**
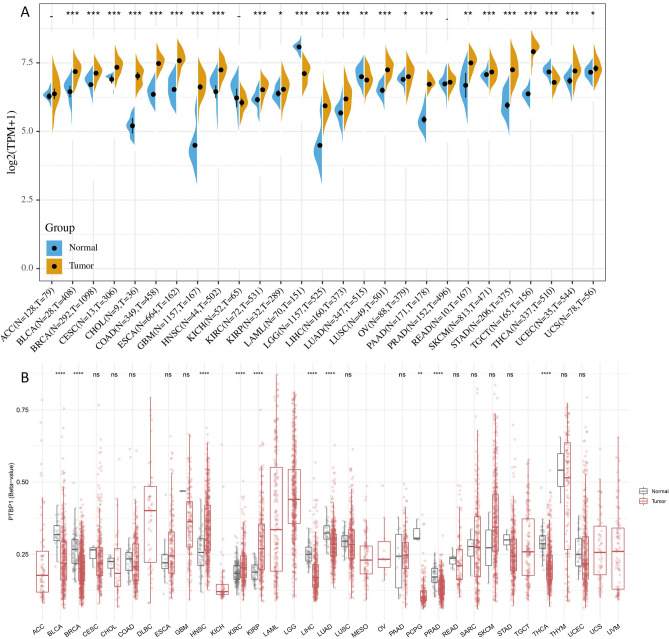
The expression and methylation of PTBP1 in various cancers. (**A**) Differences in the expression of PTBP1 between tumor tissues and normal tissues of 33 cancers in the TCGA + GTEx database. (**B**) Comparison of mean PTBP1 gene methylation in tumor tissues and normal tissues in 33 cancers from the SMART database. A t-test for comparing means between groups.

In order to determine the difference in PTBP1 expression between tumors and normal tissues, the TCGA database combined with the GTEx database was used to analyze PTBP1 mRNA levels in tumors and normal tissues of various cancer types. The analysis showed that PTBP1 expression is significantly higher in BLCA, BRCA, CESC, CHOL, COAD, ESCA, GBM, HNSC, KIRC, LGG, LIHC, LUSC, PAAD, SKCM, STAD, TGCT, UCEC in tumor tissues (P < 0.001, Fig. 1A), but significantly lower in LAML, LUAD, and THCA. These results indicate that PTBP1 is involved in regulating gene expression in the occurrence and development of most cancers. Besides, through the SMART (http://www.bioinfo-zs.com/smartapp/) database, the methylation patterns of 33 common cancers were explored. The results showed the average beta value of PTBP1 gene methylation sites decreased significantly in BLCA, BRCA, LIHC, LUAD, PCPG, PRAD, THCA, and increased significantly in HNSC, KIRC, and KIRP (P < 0.001, Fig. 1B). Overall, the combined analysis of PTBP1 expression and methylation patterns suggests that PTBP1 may have a significant role in the regulation of gene expression and the development of various cancer types.

### The relationship between PTBP1 and DNA mismatch-repair genes and methyltransferase-related gene expression

**Figure 2:**
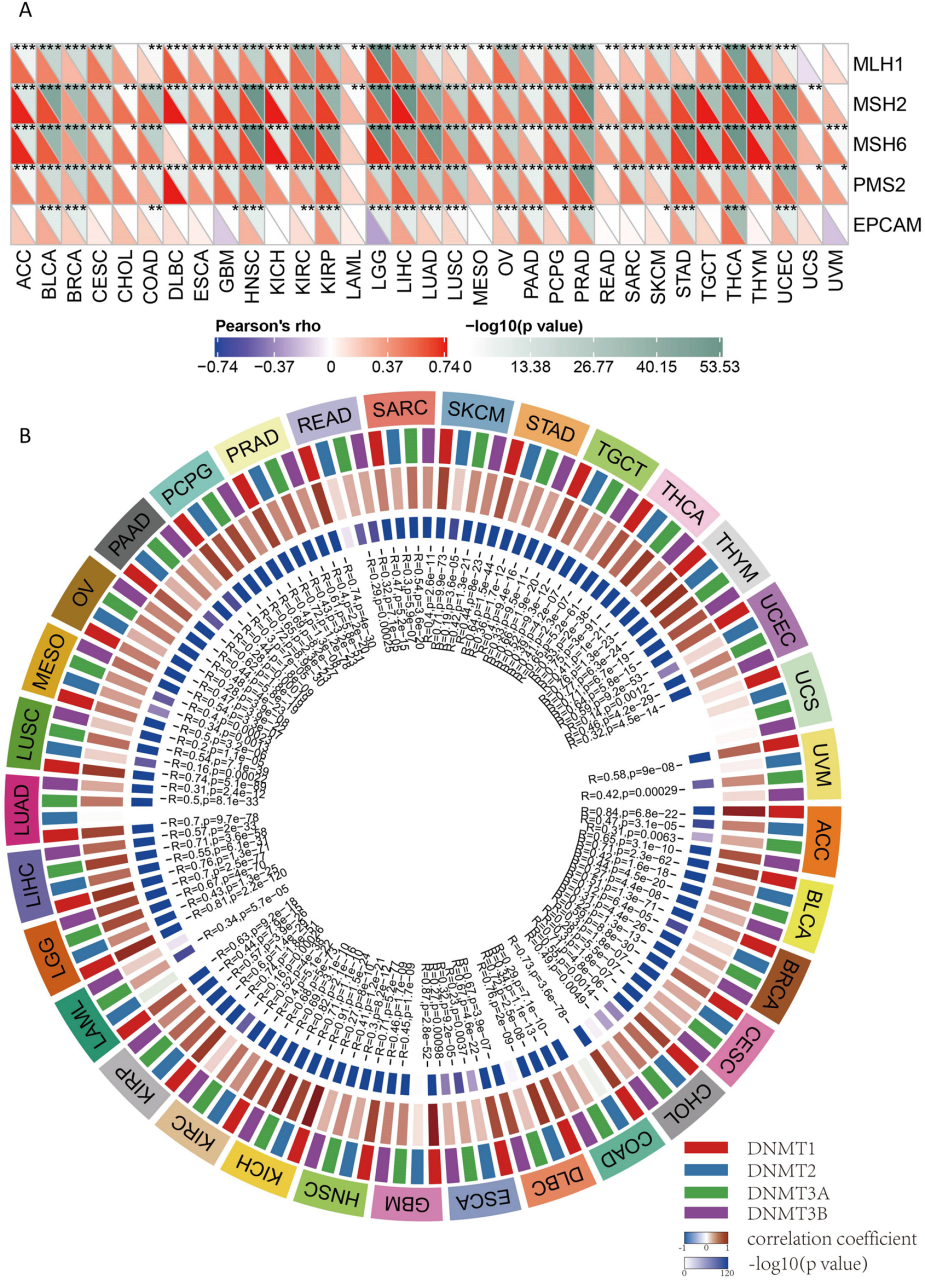
The relationship between PTBP1 and DNA repair-related genes and methyltransferase-related gene expression. (**A**) Correlation analysis between DNA repair-related genes (MLH1, MSH2, MSH6, PMS2, EPCAM) and PTBP1 expression. (**B**) Correlation analysis between DNA methyltransferase related genes and PTBP1 expression.

DNA mismatch repair is an important regulatory mechanism for maintaining the stability of the cellular genome. Loss of function of key genes involved in this mechanism results in the failure of the DNA repair machinery and cause a high degree of somatic mutation^36^. We compared the correlation between five mismatch repair-associated genes (MLH1, MSH2, MSH6, PMS2, EPCAM) and PTBP1 expression in the TCGA database (Pearson correlation, Fig. 2A). The results showed that these five mismatch repair-related genes were positively correlated with PTBP1 expression in most cancers (p < 0.01). This result suggests that the upregulation of PTBP1 is accompanied by the activation of mismatch repair-related mechanisms. This linkage effect was present in most cancers.

DNA methylation is a type of DNA chemical modification, which can affect gene expression without changing the DNA sequence^37^. DNA methylation is the covalent bonding of a methyl group to the 5'carbon position of the cytosine of the genomic CpG dinucleotide under the action of DNA methyltransferase^38,39^. Here, we analyzed the relationship between gene expression and the expression of four methyltransferases (DNMT1: red, DNMT2: blue, DNMT3A: green, DNMT3B: purple) (Fig. 2B). The results showed that PTBP1 expression had a significant positive correlation with the expression of the four genes encoding methyltransferases (P < 0.001). This result suggests that the increased expression of PTBP1 is associated with increased methylation modification activity in tumour tissues.

In summary, our analysis suggests that PTBP1 upregulation is linked to the activation of mismatch repair-related mechanisms and increased methylation modification activity in tumor tissues. These findings provide insights into the potential regulatory roles of PTBP1 in DNA repair and methylation processes in cancer.

### PTBP1 expression in relation to tumor neo-antigen, tumor mutation burden and drug sensitivity

**Figure 3:**
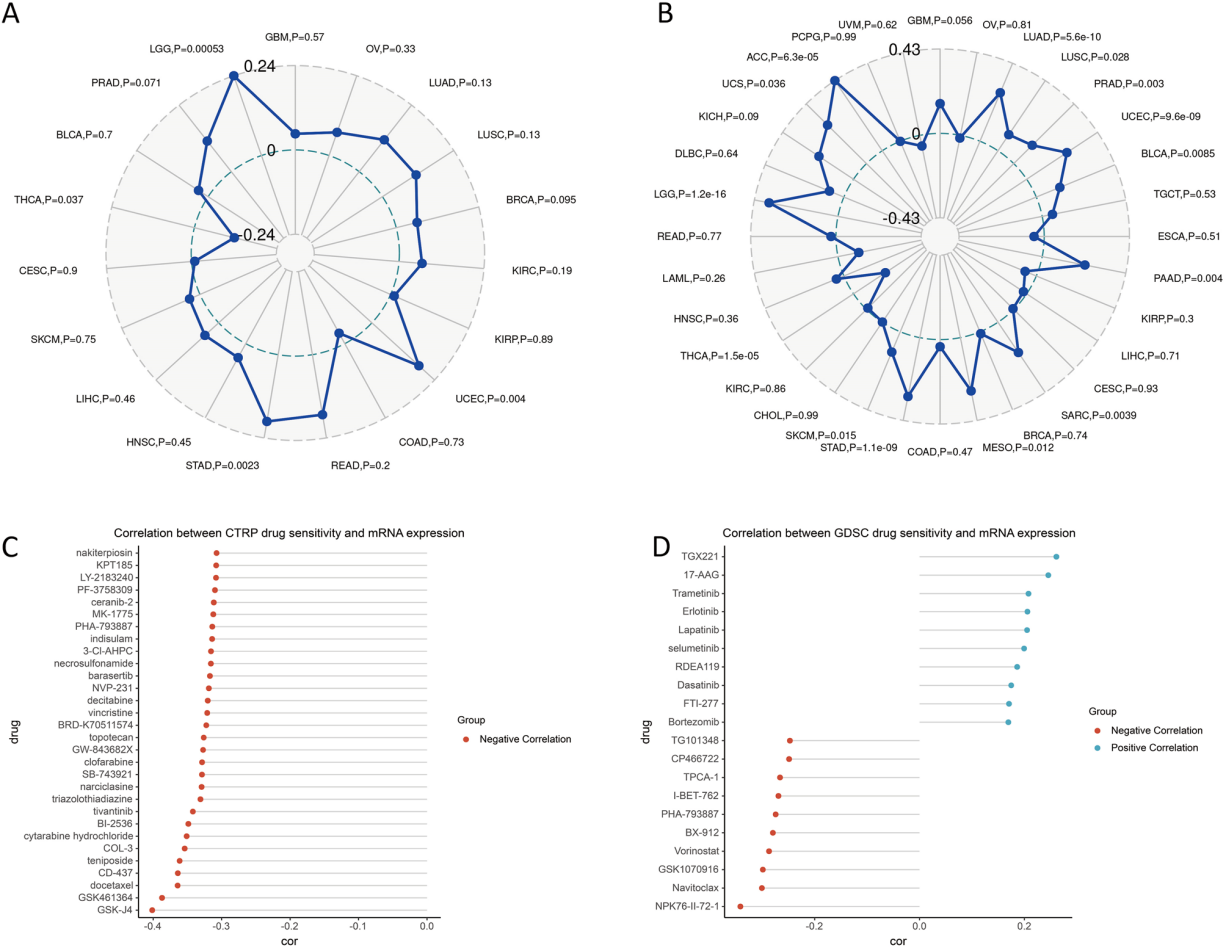
Relationship between PTBP1 expression and neo-antigens, tumor mutation burden and drug sensitivity. (**A**) The Spearman correlation between the number of tumor neo-antigens and the expression of PTBP1 in 33 cancers in the TCGA database. (**B**) The Spearman correlation analysis between the number of tumor mutations and the expression of PTBP1 in 33 cancers in the TCGA database. (**C**) The top 30 drugs in the CTRP database that were significantly correlated with PTBP1 expression. Most drug sensitivities were negatively correlated with PTBP1 expression. (**D**) The top 20 drugs significantly correlated with PTBP1 expression in the GDSC database, including 10 positively correlated drugs and 10 negatively correlated drugs.

Tumour neo-antigens are antigens encoded by mutated genes in tumour cells, mainly new abnormal proteins produced by single nucleotide mutations, deletion mutations, gene fusions, etc. These proteins can form polypeptide fragments after protease hydrolysis. These polypeptides can be presented to T cells by dendritic cells (DCs) as antigens, thereby contributing to specific immune responses mediated by T cells^40,41^. The Spearman correlation coefficient between PTBP1 gene expression and tumor neo-antigen counts was calculated (Fig. 3A). The result showed the number of tumor neo-antigens produced by LGG, UCEC, and STAD is positively correlated with the expression of PTBP1, and the opposite is true in THCA.

Tumor mutation burden (TMB) is usually measured by the number of somatic mutations (non-synonymous mutations) in the exon region of tumor cell genes on an average of 1 Mb^42^. The mutation types mainly include single nucleotide variance (SNV) and small fragment insertion/deletion (Indel), and other forms of mutations. TMB is used to reflect the number of mutations contained in tumour cells and is a quantifiable biomarker^43,44^. The Spearman correlation coefficient of PTBP1 and TMB was analyzed (Fig. 3B). The results showed that TMB in ACC, LGG, STAD, MESO, SARC, PAAD, UCEC, and LUAD was positively correlated with the expression of PTBP1, whereas in THCA, the correlation was negative. 

Chemotherapy remains one of the main treatment options for cancer. In the CTRP database, by calculating the correlation between PTBP1 expression and the IC50 of 481 small molecule drugs, it was found that the IC50 of most drugs was negatively correlated with PTBP1 expression (Fig. 3C). The result suggests that PTBP1 expression at the pan-cancer level may inhibit drug resistance in tumour cells. In the GDSC database, by the same analysis, we found that the IC50 of a large number of drugs still correlated negatively with the expression of PTBP1 (Fig. 3D, Appendix).

In summary, our analyses revealed several associations involving PTBP1 expression. PTBP1 expression was positively correlated with tumor neo-antigen counts in LGG, UCEC, and STAD, while it exhibited a negative correlation in THCA. TMB showed a positive correlation with PTBP1 expression in ACC, LGG, STAD, MESO, SARC, PAAD, UCEC, and LUAD, but a negative correlation in THCA. Additionally, PTBP1 expression displayed a negative correlation with drug resistance, as indicated by the IC50 values of various small molecule drugs in both the CTRP and GDSC databases. These findings provide insights into the potential role of PTBP1 in tumor immunology, mutational processes, and chemotherapy response across different cancer types.

### PTBP1 is implicated in the expression of immune checkpoint genes in a variety of cancers

**Figure 4:**
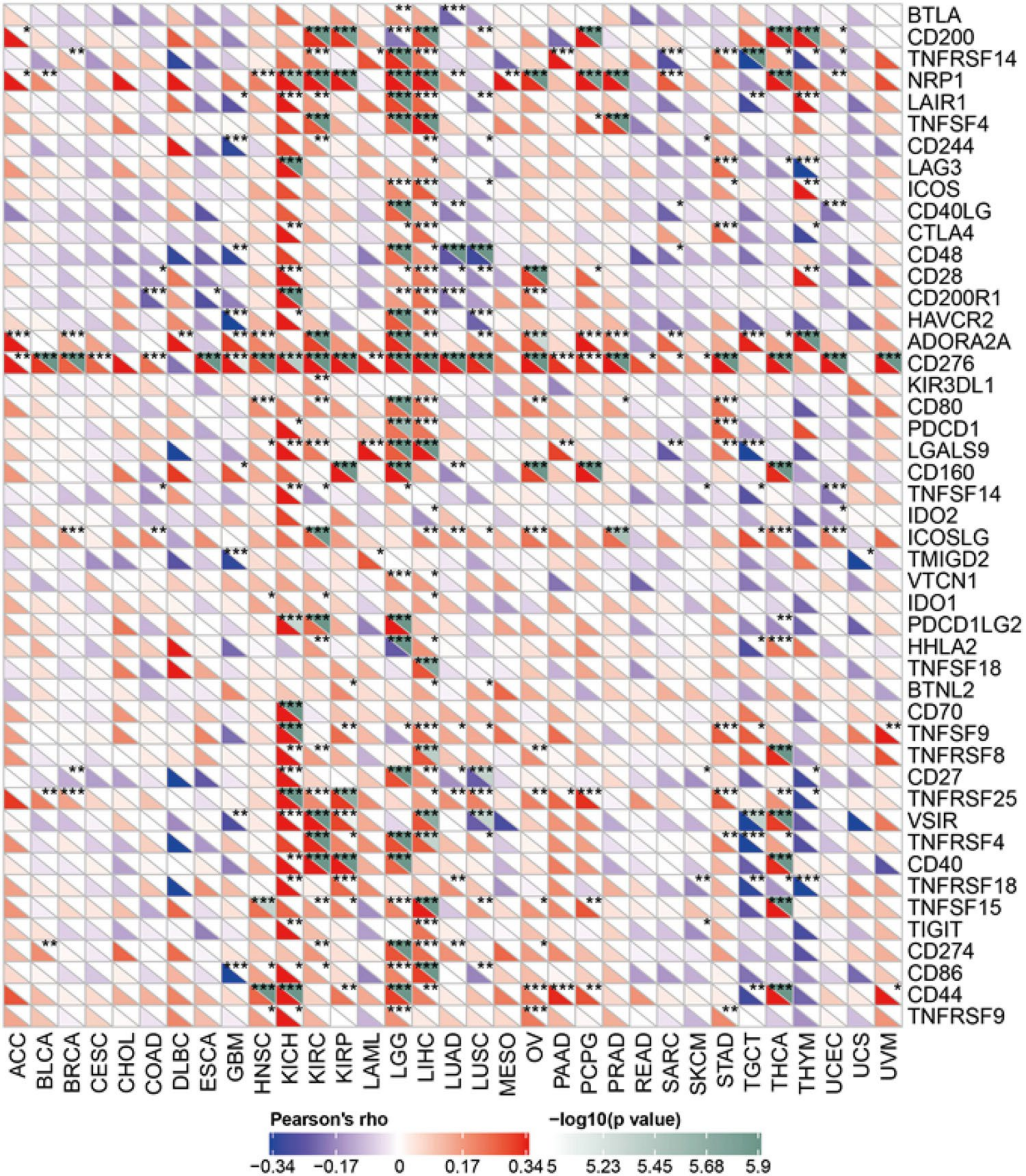
Relationship between PTBP1 gene expression in various types of tumors and immune checkpoint-related genes.

The immune system can recognize and eliminate tumor cells in the tumor microenvironment. However, immune cells are often reduced or lost in their original function in the tumour microenvironment. Immune checkpoint-based therapies have been widely used in oncology with promising results^45^. We obtained 47 immune checkpoint genes from the TCIA^46^ database and extracted the expression profile matrix of these genes from the TCGA database. Finally, the correlation of these genes with PTBP1 in different cancers was compared using the Pearson coefficient (Fig. 4). The results showed PTBP1 is highly positively correlated with immune checkpoint genes in cancers such as KICH, LGG, and LIHC, while in THYM, the correlation is negative.

### Prognostic analysis of PTBP1 gene in pan-cancer

In the analysis of cancer prognosis, we utilized the univariate Cox regression model to examine the relationship between PTBP1 expression and disease-specific survival (DSS) across 33 different cancers in the TCGA dataset. To account for non-tumor-related deaths, we focused specifically on DSS and excluded LAML cases from the analysis (Table 1). We identified cancer types in which PTBP1 expression significantly influenced patient DSS for further investigation.

**Table 1:** PTBP1 pan-cancer single gene disease special survival (DSS) analysis.

Cancer	p.value	HR	95%CI_lower	95%CI_upper	SampleSize
GBM	0.638	1.07	0.801	1.437	147
OV	0.945	0.99	0.771	1.274	349
LUAD	0.002	1.91	1.266	2.885	465
LUSC	0.618	1.12	0.719	1.743	442
PRAD	0.014	32.70	2.016	530.557	493
UCEC	0.610	0.89	0.570	1.392	539
BLCA	0.274	1.25	0.836	1.879	392
TGCT	0.577	0.59	0.091	3.806	134
ESCA	0.838	0.93	0.468	1.852	160
PAAD	0.175	1.55	0.823	2.917	170
KIRP	0.010	2.94	1.289	6.713	281
LIHC	0.052	1.50	0.997	2.246	357
CESC	0.225	1.61	0.746	3.479	287
SARC	0.011	1.90	1.162	3.111	253
BRCA	0.985	1.01	0.570	1.773	1057
MESO	0.002	4.64	1.770	12.149	64
COAD	0.604	1.17	0.647	2.116	422
STAD	0.860	0.97	0.679	1.382	332
SKCM	0.059	1.35	0.989	1.840	446
CHOL	0.373	0.51	0.117	2.235	35
KIRC	0.181	0.77	0.528	1.128	517
THCA	0.688	1.57	0.173	14.296	495
HNSC	0.823	1.05	0.710	1.539	474
READ	0.669	1.35	0.337	5.440	153
LGG	0.000	2.86	2.092	3.917	498
DLBC	0.567	0.50	0.046	5.434	47
KICH	0.000	61.39	6.325	595.800	64
UCS	0.310	1.67	0.622	4.463	53
ACC	0.001	3.29	1.653	6.565	77
PCPG	0.155	16.30	0.346	766.698	179
UVM	0.331	1.92	0.515	7.158	80

**Figure 5:**
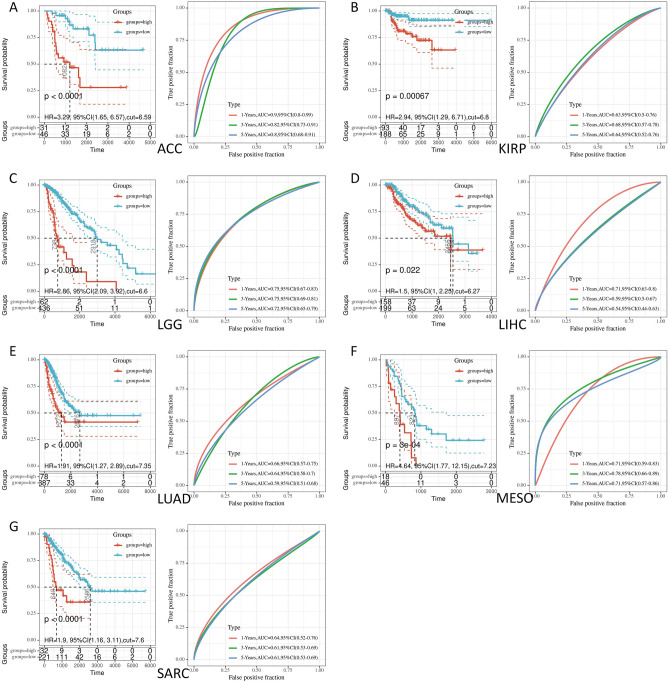
Prognostic analysis of the PTBP1 gene in pan-cancer. Among the 7 cancers, the PTBP1 high expression group and the low expression group showed significant differences in prognosis and survival (Disease Special Survival, DSS). We also predicted the 1-, 3-, and 5-year prognostic survival rates of these cancer patients based on the Cox regression model.

For the selected cancer types, we generated Kaplan–Meier survival curves to compare the survival differences between the PTBP1 high and low expression groups. The results demonstrated significant differences in DSS among seven cancers (LUAD, KIRP, LIHC, SARC, MESO, LGG, ACC) with a log-rank P-value of less than 0.001 (Fig. 5). These findings indicate that PTBP1 expression serves as a prognostic risk factor in these cancers.

Furthermore, we constructed a survival model and plotted ROC curves to assess the predictive performance of PTBP1 expression for 1-year, 3-year, and 5-year survival in the three cancer types (ACC, LGG, MESO). The analysis revealed that PTBP1 expression exhibited significant predictive power (AUC > 0.7) for prognostic survival at 1, 3, and 5 years in all three cancer types.

In summary, our analysis indicates that PTBP1 expression is a prognostic risk factor in several cancers, including LUAD, KIRP, LIHC, SARC, MESO, LGG, and ACC. The Kaplan–Meier survival curves highlight significant differences in survival between PTBP1 high and low expression groups. Additionally, the constructed survival model demonstrates that PTBP1 expression has predictive value for 1-year, 3-year, and 5-year survival in ACC, LGG, and MESO. These findings suggest that PTBP1 expression may serve as a valuable prognostic biomarker in these specific cancer types.

### PTBP1 and the tumour immune microenvironment

Tumor-infiltrating lymphocytes reflect the immune infiltration state of the tumor immune microenvironment and are highly positively correlated with cancer progression and metastasis^47^. From the previous study, we found that the expression of PTBP1 in 7 cancers is positively correlated with the prognosis of cancer patients' DSS. We included KICH in the study cohort because of the greater impact of PTBP1 expression on its prognosis (Table1). In the follow-up study, we focused on these eight cancers and studied the relationship between PTBP1 expression and tumor immune infiltration. The results of the immune assessment of all 33 cancers can be found in Fig. S1 (ESTIMATE), Fig. S2 (MCPcounter), Fig. S3 (TIMER), and Fig. S4 (CIBERSORT).

**Figure 6:**
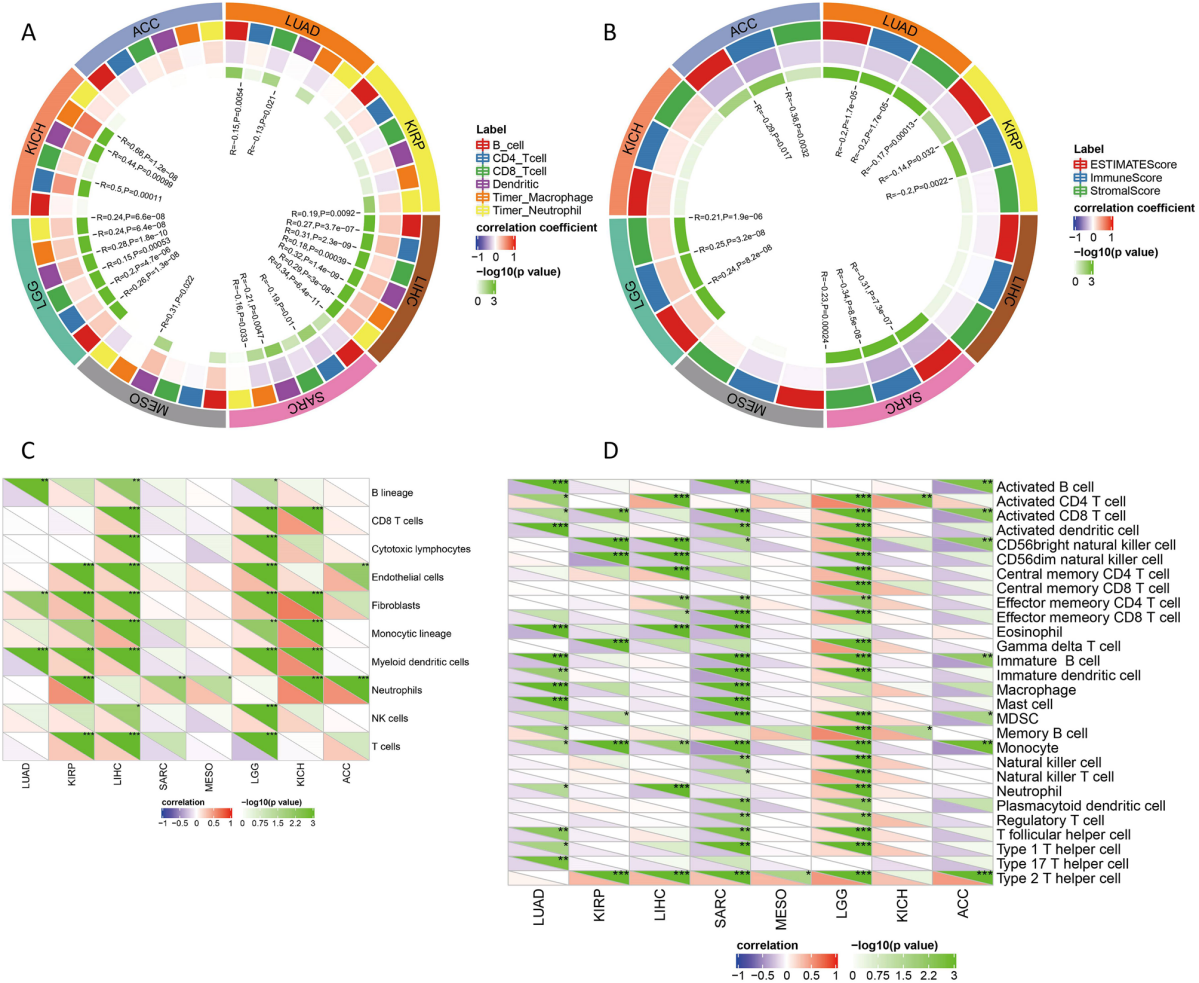
The relationship between PTBP1 and the immune microenvironment of various tumors. (**A**) The TIMER2.0 database was used to assess the immune scores of these eight cancers and the Pearson correlation coefficient was used to assess the correlation between the infiltration scores of these immune cells and PTBP1. The same analysis was performed for the immuno-infiltration score obtained by ESTIMATE (**B**), MCPcounter (**C**), and CIBERSORT (**D**). The correlation data of immune scores and PTBP1 for all 33 cancers are stored in Figs. S1–S4.

We used six immune infiltration scores obtained from TIMER2.0 and found that all six immune infiltration scores were positively correlated with PTBP1 expression in LGG and LIHC (Fig. 6A). Similarly, correlations were calculated between PTBP1 expression and three immune scores obtained by ESTIMATE (Fig. 6B), 10 immune scores obtained by MCPcounter (Fig. 6C), and 22 immune scores obtained by CIBERSORT (Fig. 6D).

From the immune infiltration scores obtained by the Cibersort algorithm, the expression of PTBP1 in LGG was positively and significantly correlated with immune-activated cells. In contrast, in SARC patients, most immune cells were negatively correlated with the expression of PTBP1. This illustrates that in LGG, increasing PTBP1 expression was accompanied by increased immune cell infiltration, whereas in SARC, the opposite was true.

Overall, our study highlights that immune cell infiltration in most cancers is associated with PTBP1 gene expression. The positive correlation between PTBP1 expression and immune infiltration in LGG and LIHC indicates a potential role for PTBP1 in modulating the tumor immune microenvironment. Conversely, the negative correlation observed in SARC suggests a distinct relationship between PTBP1 expression and immune cell infiltration in this particular cancer type. These findings provide insights into the complex interactions between PTBP1 and the tumor immune microenvironment across different cancers.

### Functional analysis of PTBP1 expression in LGG

**Figure 7:**
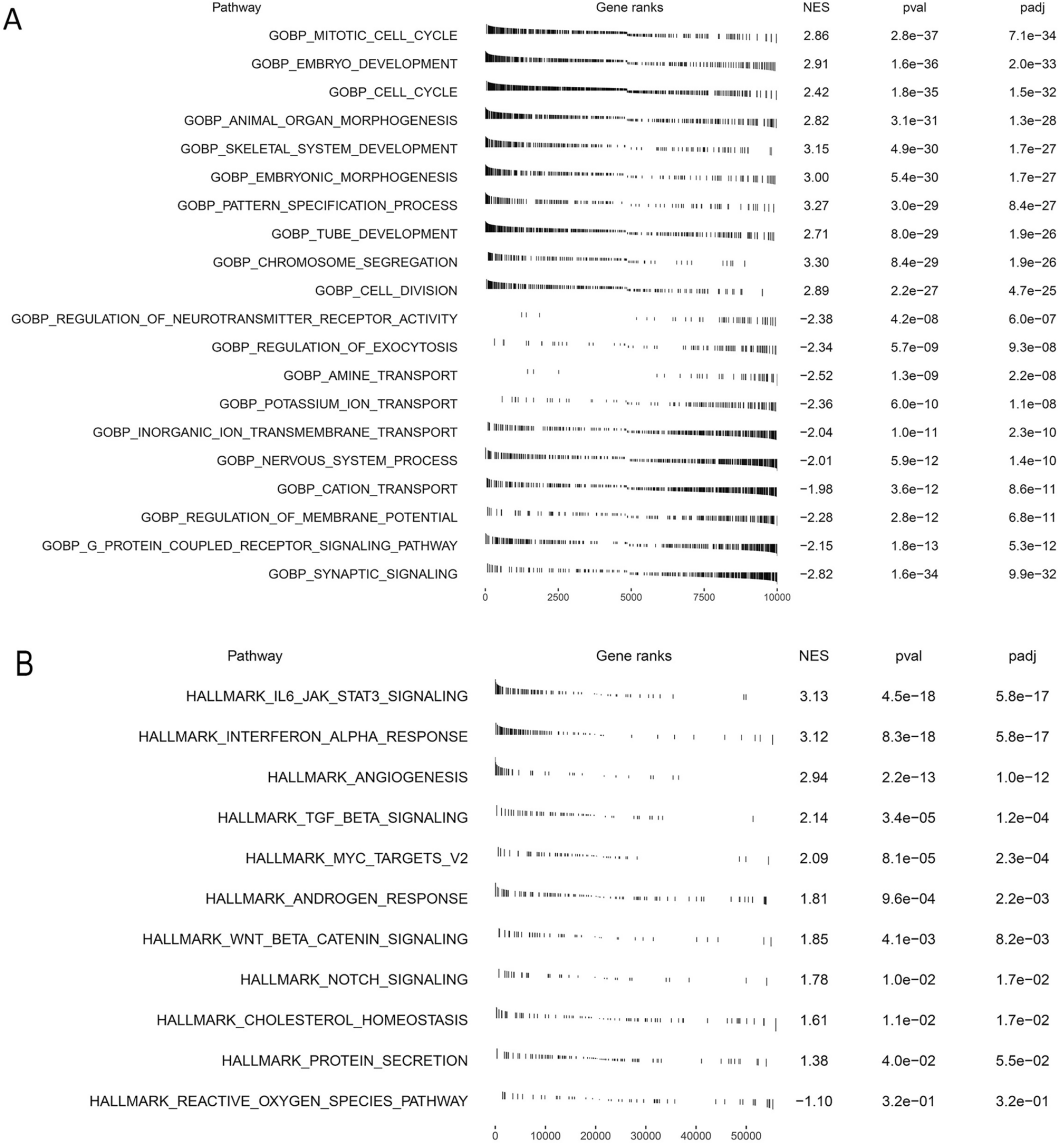
GSEA analysis of PTBP1 inTCGA LGG cohort. LGG tumor samples are divided into high expression group and low expression group according to the average expression of PTBP1. (**A**) GSEA of GO: BP gene sets. (**B**) GSEA of HALLMARK gene sets.

From the previous study, we found that the expression of PTBP1 was significant for LGG patients. To further explore the effect of PTBP1 expression on LGG patients, we divided tumor samples into high expression group and low expression group according to the average expression of PTBP1. We then analyzed the functional enrichment status of GO: BP (Fig. 7A) and HALLMARK (Fig. 7B) signal pathways using GSEA algorithms. In the low expression group, the function of the GO:BP gene sets were enriched in neurotransmitter-related signal pathways, such as synaptic signaling, G protein coupled receptor signaling pathway, regulation of membrane potential, which was strictly related to the normal function of the nervous system; while in the high expression group, the function of the GO:BP gene sets were enriched in Mitotic cell cycle, Embryo development, cell cycle signalling pathway. It shows that the high expression of PTBP1 is associated with cell cycle-related signaling pathways. The low expression of PTBP1 may indicates that the tumor cells are resting and various metabolisms are inactive; In contrast, the high expression of PTBP1 may present that the tumor cells are in an activated state, and tumor cells start to proliferate and evolve.

### Association of IDH1 mutations, tumor grade and PTBP1 expression

**Figure 8:**
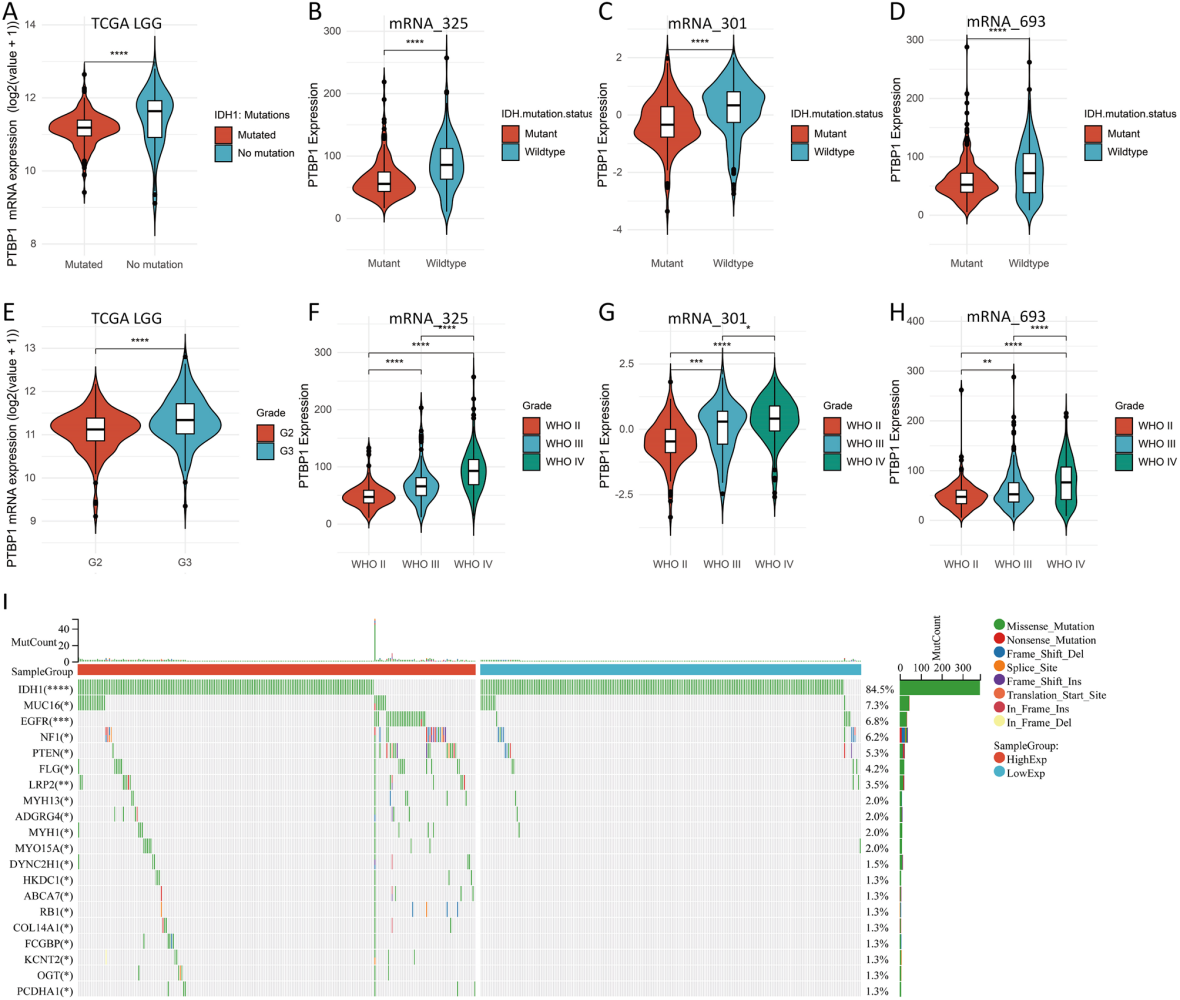
Association of PTBP1 with IDH1 mutations and the WHO grade of gliomas. (**A**) PTBP1 expression was significantly reduced in the TCGA LGG dataset in IDH1 mutant relative to wild-type LGG patients (Wilcoxon-test, P < 0.001). (**B**–**D**) Consistent findings were obtained in three separate datasets of mRNA_325, mRNA_301, mRNA_693 from the CGGA database. (**E**) Additionally, PTBP1 expression was significantly higher in Grade III compared to Grade II tumors in the TCGA LGG cohort (Wilcoxon-test, P < 0.001). (**F**–**H**) Validated in three separate datasets from CGGA. PTBP1 expression increased significantly with increasing tumour grade. In the mRNA_325, mRNA_301, mRNA_693 dataset, we used all samples from WHO II, WHO III, WHO IV. Where WHO II, WHO III graded gliomas are considered LGG, and WHO IV graded gliomas are considered GBM. (**I**) Comparison of gene mutation differences between the high and low PTBP1 expression groups in TCGA LGG cohort. Here, we selected the top 20 genes with the largest mutational differences (chi-square test, P < 0.05 was considered statistically different).

IDH1 is highly mutated in LGG and GBM and has a significant impact on the prognosis of LGG and GBM. A number of studies have demonstrated that LGG patients with IDH1 mutations have a better prognosis^48^. The pathological grading of gliomas is an important indicator of the malignancy of a glioma. According to WHO pathological grading criteria, gliomas can be classified into four grades. Of these, WHO II and WHO III are low malignancy gliomas (LGG), while WHO IV is a highly malignant glioma (GBM). In order to reveal the relationship between IDH1 mutation, tumour grade and PTBP1 expression, TCGA LGG sample cohort, CGGA mRNA_325, CGGA mRNA_301, CGGA mRNA_693 sample cohort were used to compare the difference in PTBP1 expression between IDH1 mutant and wild type, and different tumour grades of glioma patients with PTBP1 expression in patients. Figure 8A shows that in the TCGA-LGG cohort, patients with IDH1 mutations had lower PTBP1 expression compared to wild type, which was significantly different (p < 0.0001). The results can be verified in three independent cohorts, CGGA mRNA_325, CGGA mRNA_301, CGGA mRNA_693 (Fig. 8B–D). The results suggest that patients with IDH1 mutations have lower PTBP1 expression. This conclusion is consistent with previous analyses considering that high PTBP1 expression is a poor prognostic factor in LGG patients. To demonstrate the relationship between glioma grade and PTBP1 expression in the TCGA LGG cohort, we compared the difference in PTBP1 expression between Grade2 and Grade3 (Fig. 8E). The results showed that the expression of PTBP1 was significantly higher in Grade3 than in Grade2. We further validated this result in three cohorts of CGGA (Fig. 8F mRNA_325, Fig. 8G mRNA_301, Fig. 8H mRNA_693). We found that as tumour malignancy increased, the expression of PTBP1 likewise increased significantly. The results demonstrate that increased PTBP1 is an important molecular marker of glioma progression. To further demonstrate the prognostic impact of PTBP1, we validated the relationship between PTBP1 expression and glioma (LGG/GBM) prognosis in three separate datasets, CGGA mRNA_325,CGGA mRNA_301, and CGGA mRNA_693, respectively. The results demonstrated that PTBP1 expression was a poor prognostic factor in all three datasets (Figs. S5–S7). Taken together, PTBP1 expression is influenced by IDH1 mutations and the degree of tumour malignancy, and is associated with poor prognosis in gliomas.

Furthermore, we conducted a comparison of the mutational profiles between the PTBP1 high and low expression groups. We identified the top 20 genes that exhibited significant differences (Fig. 8I). Interestingly, we observed that IDH1 mutations were less prevalent in the PTBP1 low expression group, while the other 19 genes showed the opposite pattern. It is noteworthy that although the majority of mutational events in LGG patients were associated with the IDH1 wild type, the PTBP1 low expression group displayed a higher number of unique mutations in genes such as MYO15A, DYNC2H1, HKDC1, ABCA7, RB1, COL14A1, FCGBP, KCNT2, OGT, and PCDHA1. These findings suggest that PTBP1 expression is closely associated with the mutational landscape of LGG.

### Methylation site analysis of PTBP1

**Figure 9:**
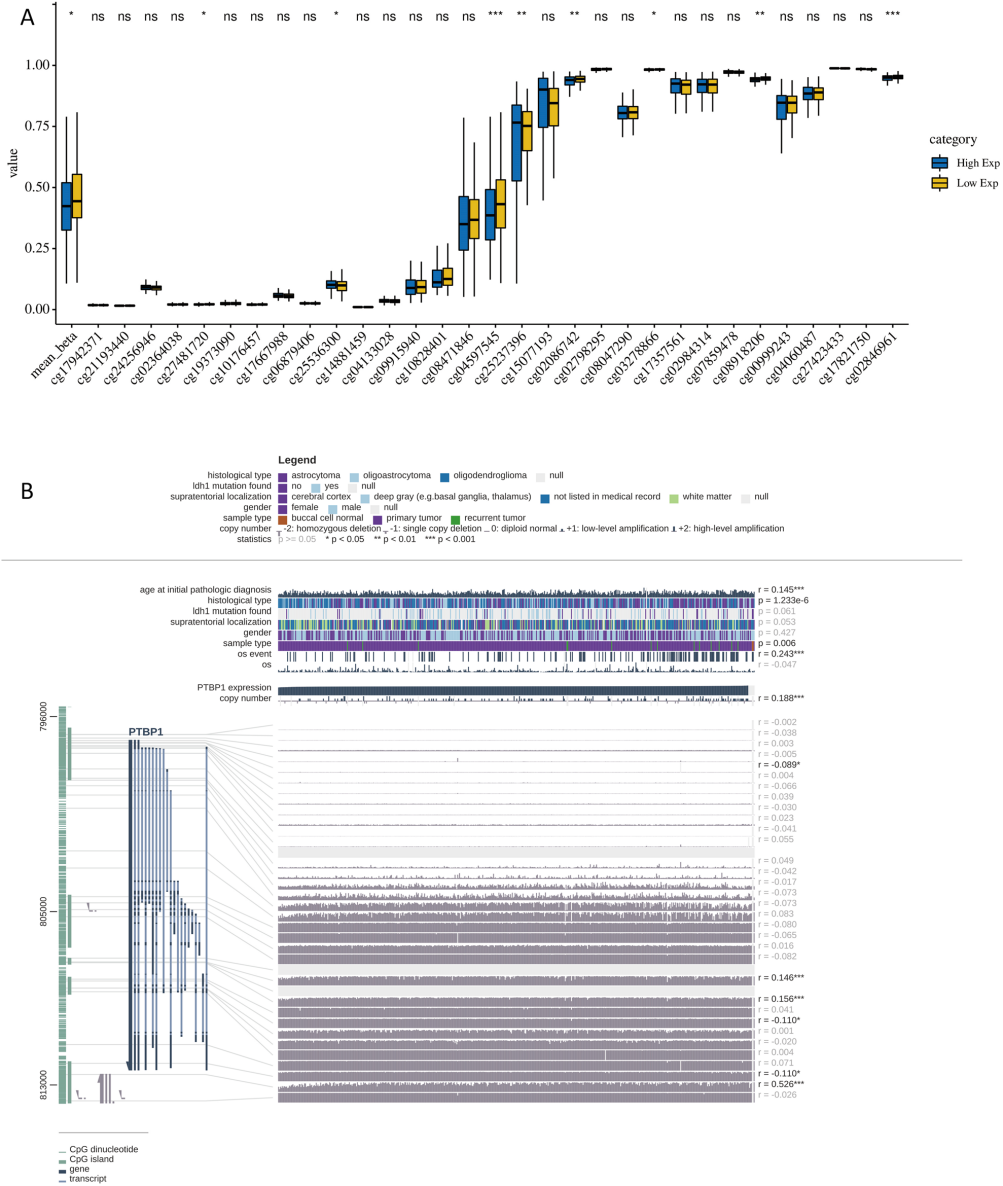
Correlation analysis between PTBP1 expression and methylation sites in TCGA LGG cohort. (**A**) The methylation sites of PTBP1 gene differed in the methylation of PTBP1 high expression group and low expression group. (**B**) Ranking of beta values and clinical information of methylation sites of PTBP1 gene based on PTBP1 gene expression data. Categorical variables were compared using ANOVA, while the Spearman correlation coefficient was employed to analyze the correlation between continuous variables and PTBP1 expression.

**Figure 10:**
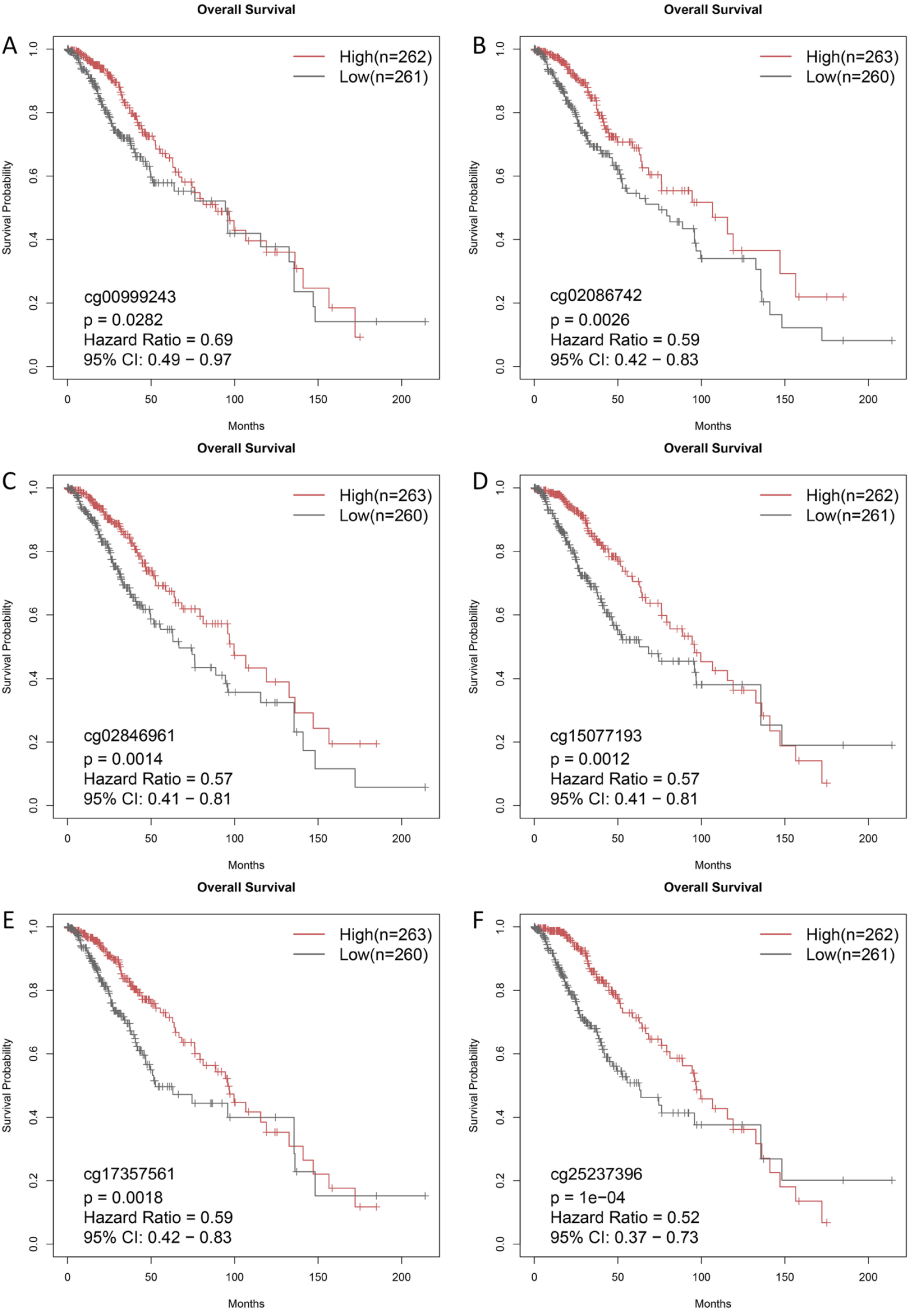
Relationship between the beta-value of methylation sites and the prognostic OS of patients. Here we divided the LGG samples into hypermethylation group and hypomethylation group according to the mean beta-value of each methylation site. The Kaplan–Meier survival curve was used to evaluate the difference between the two groups. Here we only show the methylation sites with significant differences in survival curves (Hazard Ratio < 1, P < 0.05, **A**–**F**).

Generally, methylation modification of genes can reduce the transcriptional expression of genes. We obtained beta values for 31 methylation sites of PTBP1 from the SMART database. Grouped according to the PTBP1 expression level and compared the differences in the degree of methylation of 31 methylation sites between the two groups (T-test). The results showed that the degree of methylation in the high expression group of PTBP1 was significantly lower than that in the low expression group (P < 0.05). Among them, cg027481720, cg25536300, cg04597545, cg25237396, cg02086742, cg03278866, cg08918206, and cg02846961 showed significant differences (Fig. 9A). To further reveal the correlation between PTBP1 expression and the degree of methylation of its methylation sites and clinical traits (age at initial pathologic diagnosis, histological type, IDH1 mutation found, supratentorial localization, gender, sample type, os event, os), Mexpress (https://mexpress.be/) database to be used to retrieve methylation data for PTBP1 in the TCGA LGG cohort. As a result, we found that age at diagnosis of LGG showed a weak positive correlation with PTBP1 expression (Spearman, r = 0.145, P < 0.001). The mortality events of the patients showed a significant positive correlation with PTBP1 expression (Spearman, r = 0.243, P < 0.001), while the survival time of the patients was not related to the expression of PTBP1. This result suggests that high PTBP1 expression is likely to be a driver of cancer progression. In addition, we found that methylation at the cg24840300 site downstream of PTBP1 was significantly and positively correlated with PTBP1 expression (r = 0.526, P < 0.001). This site may serve as an important target for drug action in regulating PTBP1 gene expression (Fig. 9B). We calculated the correlation between the expression of PTBP1 and its 31 methylation sites to find the critical methylation sites that regulate the expression of PTBP1 (Fig. S8). The results showed that the methylation of cg10828301, cg04597545, and cg08918206 would significantly reduce the mRNA expression of PTBP1, while the methylation of cg15077193, cg17357561, and cg17821750 would significantly increase the mRNA expression of PTBP1 (P < 0.01, R > 0.1). The Univariable Cox proportional regression was used to analyze the methylation sites and patients' overall survival (Table 2). Kaplan–Meier survival analysis was used to calculate the difference between the degree of methylation at the methylation site (based on the mean value) and the patient’s prognostic OS. The results showed that the degree of methylation of cg00999243, cg02086742, cg02846961, cg15077193, cg17357561, and cg25237396 was significantly related to the prognostic survival of patients, and the hyper-methylation of these sites was beneficial to the prognostic OS of patients (P < 0.05, HR < 1.0) (Fig. 10).

**Table 2:** Overall survival analysis of the beta-value of 31 methylation sites in PTBP1 gene in the TCGA LGG cohort.

Meth_Site	p.value	HR	95%CI_lower	96%CI_upper
cg17942371	0.128	0.760	0.54	1.08
cg21193440	0.315	0.840	0.6	1.18
cg24256946	0.302	0.840	0.6	1.18
cg02364038	0.790	0.960	0.68	1.34
cg27481720	0.580	0.910	0.65	1.28
cg19373090	0.872	1.030	0.73	1.44
cg10176457	0.616	0.920	0.65	1.29
cg17667988	0.159	1.280	0.91	1.8
cg06879406	0.970	1.010	0.72	1.41
cg25536300	0.358	1.170	0.83	1.65
cg14881459	0.142	0.770	0.55	1.09
cg04133028	0.962	1.010	0.72	1.42
cg09915940	0.839	1.040	0.74	1.46
cg10828401	0.531	0.900	0.64	1.26
cg08471846	0.141	0.780	0.55	1.09
cg04597545	0.094	0.750	0.53	1.05
cg25237396	0.000	0.520	0.37	0.73
cg15077193	0.001	0.570	0.41	0.81
cg02086742	0.003	0.590	0.42	0.83
cg02798295	0.727	0.940	0.67	1.32
cg08047290	0.088	0.750	0.53	1.05
cg03278866	0.505	1.120	0.8	1.58
cg17357561	0.002	0.590	0.42	0.83
cg02984314	0.191	0.800	0.57	1.12
cg07859478	0.980	1.000	0.71	1.4
cg08918206	0.319	0.840	0.6	1.18
cg00999243	0.690	0.028	0.49	0.97
cg04060487	0.997	1.000	0.71	1.4
cg27423433	0.110	1.320	0.94	1.86
cg17821750	0.667	1.080	0.77	1.52
cg02846961	0.001	0.570	0.41	0.81

### GSEA algorithm to analyze the function of PTBP1 methylation-related genes

**Figure 11:**
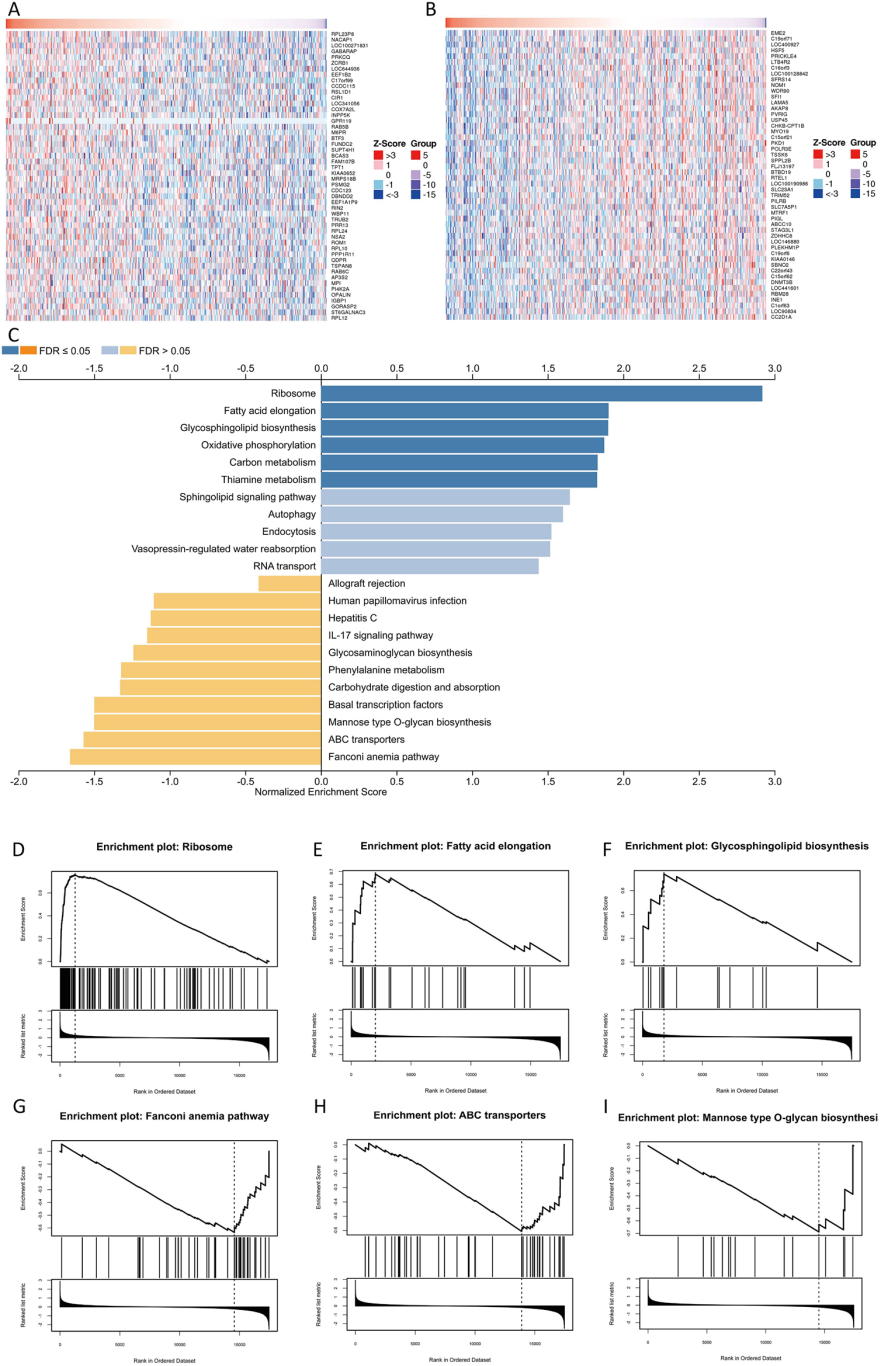
Gene set enrichment analysis of PTBP1 methylation-related genes. LinkedOmics was used to analyse PTBP1 methylation-associated genes and to rank them according to the correlation coefficient statistic. (**A**) Genes negatively associated with PTBP1 methylation in LGG patients (Spearman, P < 0.05, top50). (**B**) Genes positively associated with PTBP1 methylation (Spearman, P < 0.05, top50). (**C**) Functional enrichment analysis of PTBP1 methylation-associated genes. The PTBP1 methylation-related genes were ranked according to FDR values and the gene sets were scored using the WebGestalt database, resulting in a set of genes positively and negatively associated with the degree of PTBP1 methylation. (**D**–**F**) The top3 pathways of the set of genes positively associated with PTBP1 methylation, including Ribosome, Fatty acid elongation, Glycosphingolipid biosynthesis. (**G**–**I**) The top3 pathways of the set of genes negatively associated with PTBP1 methylation, including: Fanconi anemia pathway, ABC transporter, Mannose type O-glycan biosynthesis. pathways.

To further analyse the association of methylation of PTBP1 with LGG. We performed correlation analysis based on the mean methylation of PTBP1 with gene expression in LGG patients and performed GSEA based on the Spearman correlation coefficient as the rank value of the gene (using the MSIGDB database, Fig. 11A–C). The results showed that as the methylation of PTBP1 increased, the signalling pathways such as Ribosome, Fatty acid elongation and Glycosphingolipid biosynthesis were significantly enriched in higher fractions (p < 0.05, Fig. 11D–F). The signalling pathways such as Fonconi anemia pathway ABC transporters, Mannose type O-glycan biosynthesis were mainly enriched in the PTBP1 hypermethylated samples (p < 0.05, Fig. Fig. 11G–I). These results suggest that the regulation of methylation of PTBP1 is significantly implicated in both the initiation and development of LGG. In addition, we found that activation of inflammation-related signalling pathways was associated with hypomethylation of PTBP1 (e.g.,Allograft rejection, Hepatitis C, IL-17 signaling pathway). This suggests that inflammatory processes in LGG patients may be dependent on the hypermethylated state of PTBP1.

### Protein expression of PTBP1 correlates with the malignancy of gliomas

**Figure 12:**
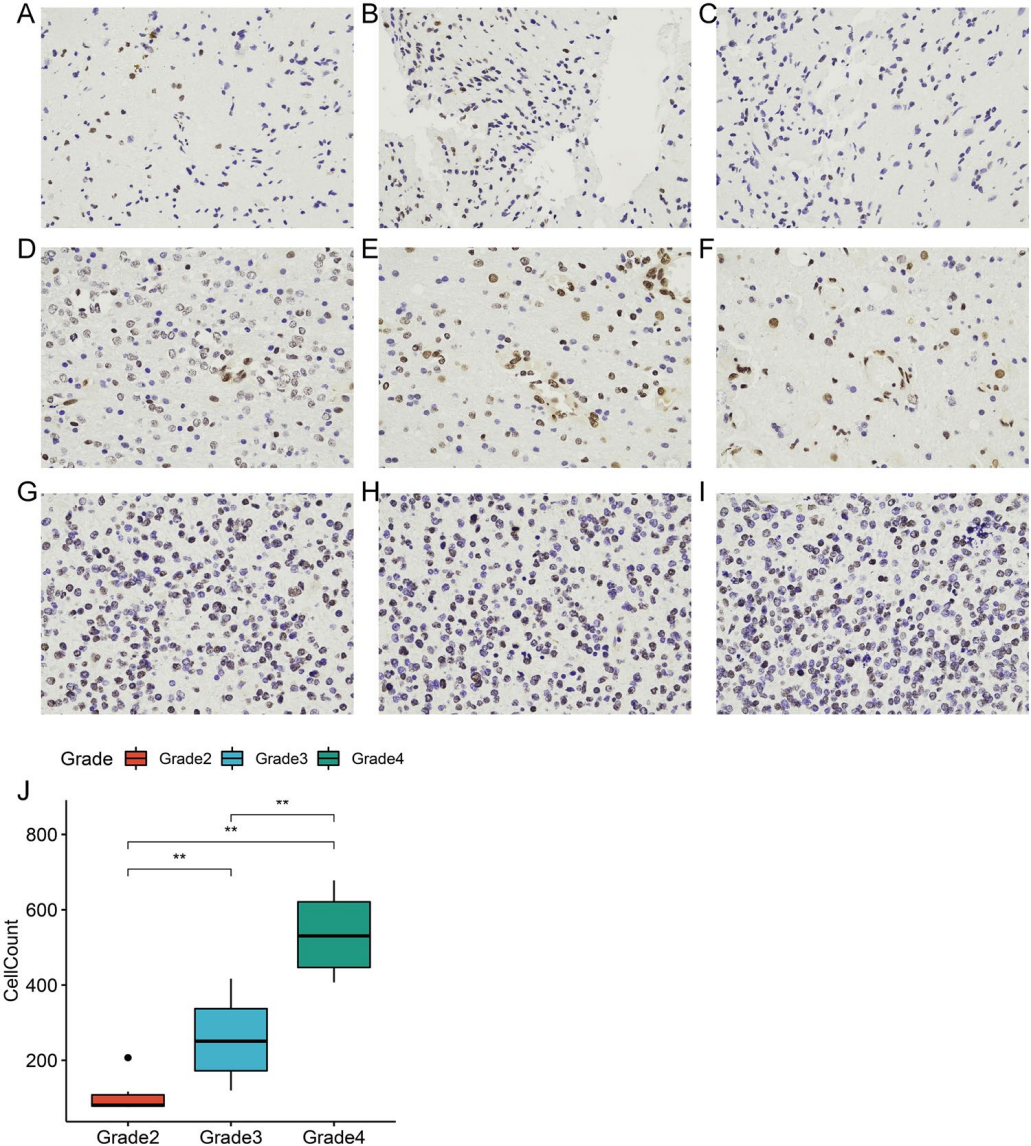
Immunohistochemical validation of the association between PTBP1 and LGG progression. (**A**–**C**) PTBP1 protein expression in LGG Grade2 patients. (**D**–**F**) PTBP1 protein expression in LGG Grade3 patients. (**G**–**I**) PTBP1 protein expression in LGG Grade4 patients. (**J**) Comparison of the number of PTBP1 + tumour cells in LGG patients with Grade2, Grade3 and Grade4 (Wilcox-test, ** P < 0.01).

In the previous study, we explored the association of PTBP1 gene expression with LGG deterioration and prognosis using public datasets. We next studied pathological sections of WHO staged Grade 2, Grade 3 and Grade 4 using immunohistochemistry. The results showed that as the malignancy of gliomas increased, more malignant cells expressing PTBP1 protein became available (Fig. 12A–I). We compared the number of PTBP1 + malignant cells in each of the three groups by Wilcox-test. The results showed that the number of PTBP1 + cells:Grade4 > Grade3 > Grade2. All three groups were significantly different from each other by two comparisons (Fig. 12J).

### PTBP1 as a non-independent prognostic indicator for glioma

**Figure 13:**
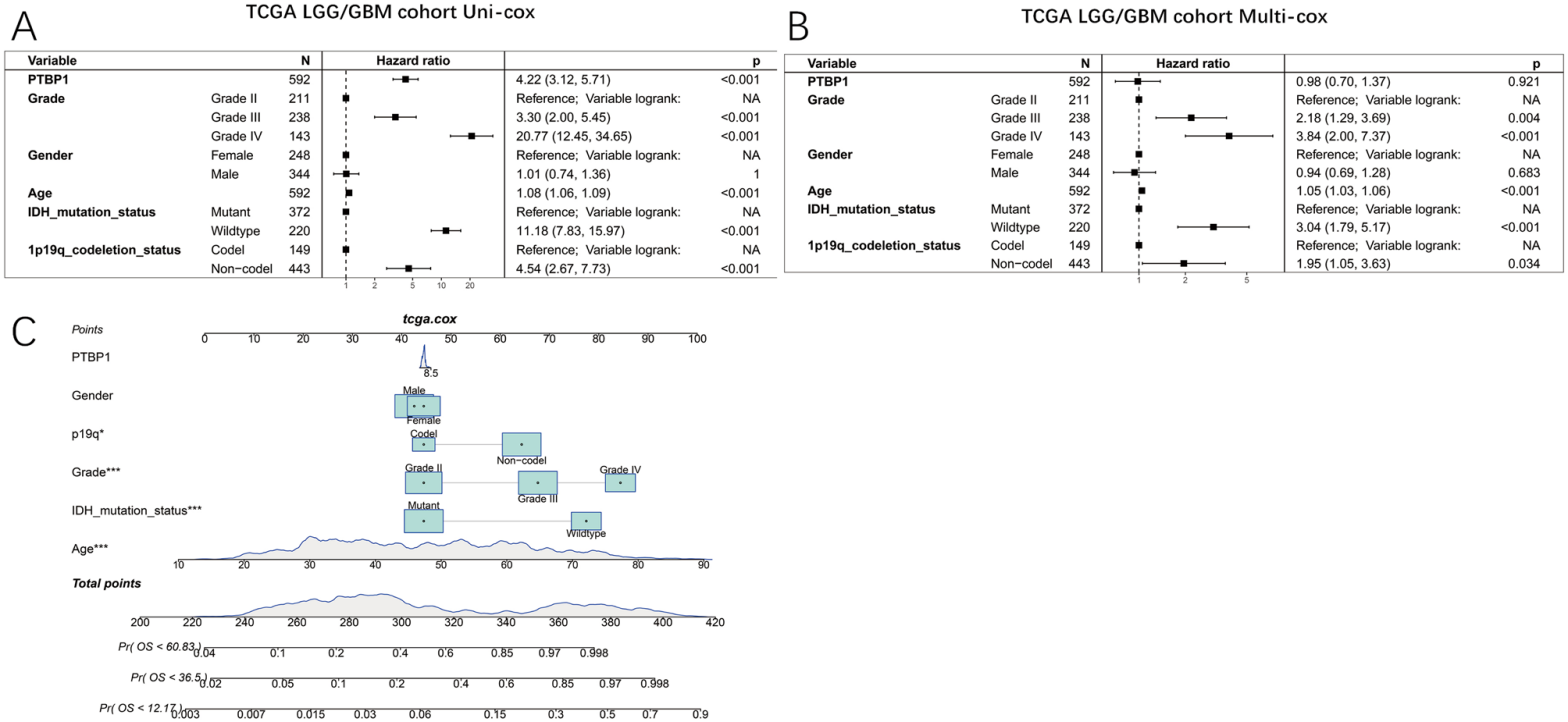
Impact of PTBP1 expression in the TCGA LGG/GBM cohort on the prognosis of glioma. (**A**) Uni-cox forest plot. (**B**) Multi-cox forest plot. (**C**) Nomogram of CGGA mRNA_301 cohort.

The previous analysis has demonstrated that high PTBP1 expression is a poor prognostic factor in LGG patients. To demonstrate more rigorously the relationship between PTBP1 and glioma prognosis, we constructed uni-cox and multi-cox regression models and generated nomograms by considering the factors of PTBP1 expression, age, gender, tumour WHO grade, IDH1 mutation, and 1p19q codeletion as variables affecting glioma prognosis. The analysis was performed in four datasets, TCGA-LGG/GBM (592 samples), CGGA mRNA_301 (279 samples), CGGA mRNA_325 (304 samples), and CGGA mRNA_693 (542 samples), respectively. The results showed that PTBP1 was not an independent prognostic factor in the TCGA-LGG/GBM cohort and the CGGA mRNA_301 cohort (Fig. 13A–C, Fig. S9). It may be due to the high correlation of PTBP1 expression with WHO classification and IDH1 mutation, both of which are independent prognostic factors for glioma prognosis (HR > 1, P < 0.001). In contrast, PTBP1 expression was an independent prognostic factor in both CGGA mRNA_325 and CGGA mRNA_693 cohorts (HR > 1, P < 0.001, Fig. S9). In these two cohorts the Harzard ratio for PTBP1 was just between 1.0 and 1.1. The results of these studies suggest that the main reason for PTBP1 as a prognostic risk factor is that PTBP1 can indicate glioma progression and IDH1 mutation status.

### Single-cell transcriptome and spatial transcriptome to explore the implication of PTBP1 in glioma

**Figure 14:**
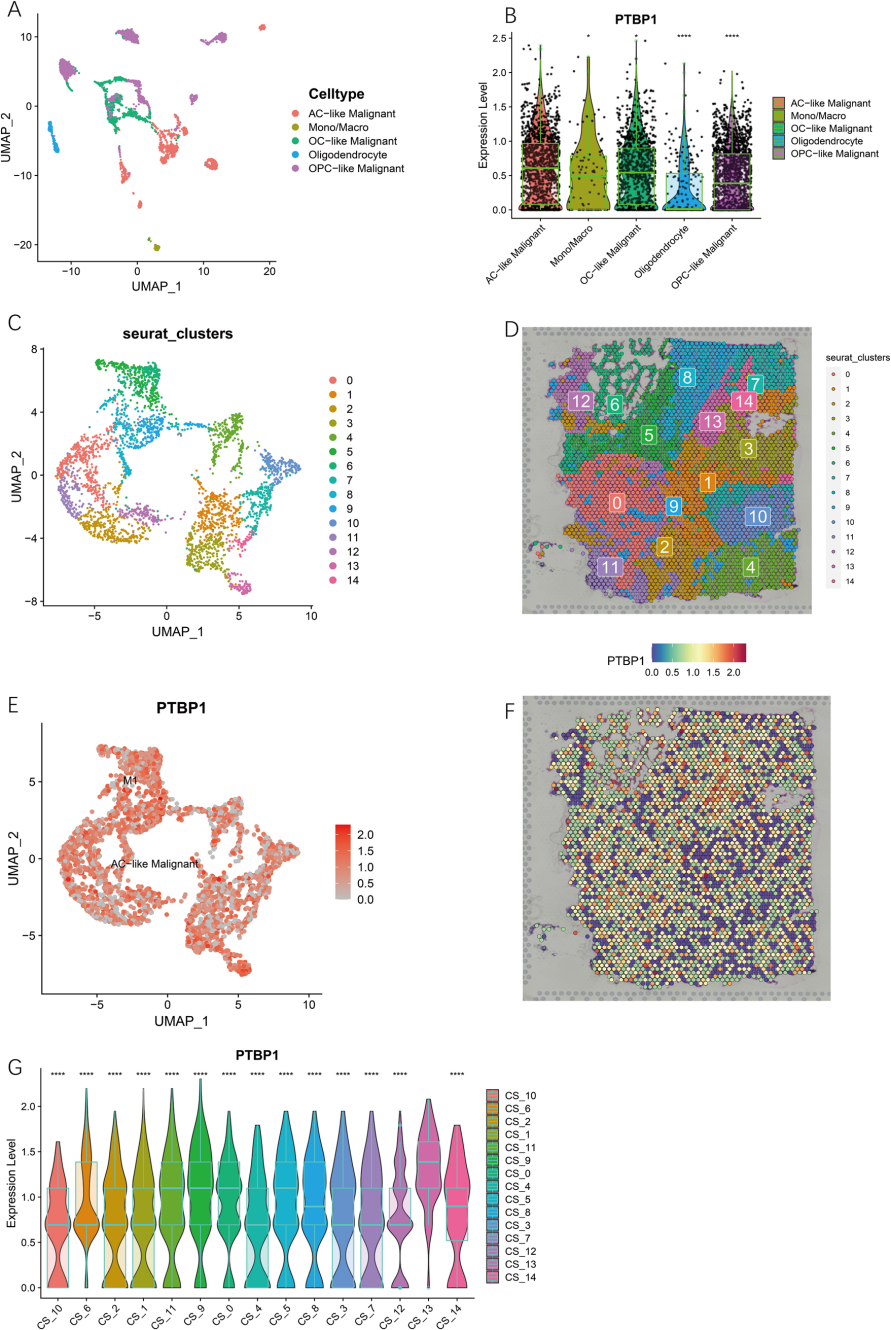
Expression of PTBP1 in the single cell transcriptome and spatial transcriptome of glioma. (**A**,**B**) PTBP1 expression is highest in AC-like Maligant in the GSE102130 dataset. (**C**,**D**) Reduced dimensional clustering and annotation of the glioma spatial transcriptome. Fifteen clusters could be obtained for 3468 transcript loci in gliomas. The vast majority of these cells were AC-like Malignant (**E**,**F**) There was no significant heterogeneity in the distribution of PTBP1 in gliomas overall. (**G**) PTBP1 expression was highest in gliomas in CS_13.

In addition to analyzing PTBP1 expression at the molecular level, we conducted cellular localization studies of PTBP1 expression using the GSE102130 dataset. Our findings revealed that PTBP1 was expressed in the majority of cells and did not exhibit significant cell specificity (Fig. 14A). Immunohistochemical analysis of glioma sections further confirmed that PTBP1 was uniformly distributed and did not show significant heterogeneity across the tumor (Fig. 12A–I).

To investigate the expression of PTBP1 in different cell types within gliomas, we compared its expression levels in four cell types, with AC-like Malignant (astrocytic differentiation) serving as the reference. The results demonstrated significant differences in PTBP1 expression between AC-like Malignant and the other cell types, including OC-like Malignant (oligodendrocytic differentiation), OPC-like Malignant (oligodendrocyte precursor cells), and Oligodendrocyte, Mono/Macro (Fig. 14B). This finding is consistent with the notion that the progression of gliomas involves a transition from oligodendrocytes to OPC-like Malignant cells and ultimately to OC-like Malignant cells. Therefore, PTBP1 expression at the single-cell level serves as an indicator of the progression of glioma cancer cells.

We also explored the spatial transcriptome of PTBP1 expression in gliomas. By analyzing 3468 transcript loci, we identified 15 clusters (Fig. 14C,D). The majority of cells belonged to the AC-like Malignant cluster, with the exception of CS_5 and CS_8, which contained a small number of M1 cells (Fig. 14E). PTBP1 expression did not exhibit a clear pattern of distribution in the UMAP scatter plots and spatial pathology sections, except for CS_13, where it was significantly higher compared to other cells (Fig. 14F,G).

These findings suggest that PTBP1 expression in gliomas is not limited to specific cell types but rather is widespread throughout the tumor. While there may be some variations in PTBP1 expression levels between different cell types, its overall distribution is relatively uniform. The elevated expression of PTBP1 in CS_13 highlights its potential importance in the molecular characteristics of that specific cluster.

In conclusion, the cellular localization studies of PTBP1 expression in gliomas revealed its widespread expression across different cell types, with AC-like Malignant cells showing significant differences compared to other cell types. Furthermore, the spatial transcriptome analysis emphasized the distribution of PTBP1 expression throughout the tumor, with specific variations observed in certain clusters. These findings contribute to our understanding of the role of PTBP1 in glioma biology and provide valuable insights for future research and therapeutic development.

## Discussion

PTBP1 was recognized as an oncogene in GBM and as having an oncogenetic function in breast cancer cell lines and ovarian tumors^49^. Moreover, PTBP1 is a potent driver of brain tumor growth and invasiveness^50^. In this study, we found that PTBP1 was highly expressed in various cancers, and its methylation was significantly reduced in these cancers compared to normal tissues. These results suggest that PTBP1 may play a crucial role in the development and progression of various types of cancer, and that epigenetic modifications of its methylation may play an important role in regulating its function.

By comparing the expression of PTBP1 mRNA in each tumor sample with the expression of DNA mismatch repair genes (MMRs) and the expression of methyltransferase-related genes, we found that mismatch repair-related genes and methyltransferase-related genes were found to be significantly positively correlated with PTBP1 expression in most cancers. In eukaryotes, DNA mismatch repair maintains the stability of the genome and prevents damage to genetic material from the external environment^51^. Defects in DNA base mismatch repair mechanisms are an important cause of cancer development^52^. PTBP1 expression was significantly and positively correlated with the expression of genes associated with DNA base mismatch repair, suggesting that elevated PTBP1 expression was accompanied by a large number of genomic mutational events. Furthermore, we have demonstrated in LGG that high PTBP1 expression is accompanied by a high genomic mutation frequency. All these factors are associated with cancer progression.

Immune checkpoint blockade (ICB) therapy significantly improves overal survival (OS) in patients with advanced melanoma, non-small cell lung cancer, urothelial carcinoma (UC) and renal cell carcinoma (RCC)^53^. Tumour Mutational Burden (TMB) has been widely used to predict the efficacy of ICB and to identify the benefit of immunotherapy for patients^54–57^. A few somatic mutations in the tumour DNA produce neo-antigens that can effectively activate the anti-tumour immune response of T cells^58–60^. We have analysed PTBP1 expression in relation to tumour neoantigens and tumour mutational burden in a variety of cancers. PTBP1 expression was found to be positively correlated with tumour neo-antigens in patients with LGG, UCEC and STAD. Tumour mutational burden was significantly and positively correlated with PTBP1 expression in ACC, LGG, STAD, MESO, SARC, PAAD, UCEC and LUAD. We explored the relationship between immune checkpoints and PTBP1. Most of the immune checkpoints were found to be positively correlated with PTBP1 expression in LIHC (39/47), LGG (32/47), and KICH (27/47) (Fig. 4). These results suggest that immune cell infiltration and activation of inflammation-related signaling pathways in these three cancer types are associated with the expression of PTBP1. In addition, we found that PDCD1 and CTLA4 were also associated with PTBP1 expression in these 3 cancer types. It suggests that PTBP1 may act as a molecular marker for the efficacy of immune checkpoint therapy^61–63^. Amazingly, CD276 expression was positively correlated with PTBP1 expression in most cancers. Studies have shown that CD276 is a prognostic indicator for glioma and that high expression of CD276 predicts a poor prognosis for glioma^64^. In LGG, the relationship between PTBP1 and CD276 needs further exploration.

Furthermore, we utilized a uni-Cox regression model to examine the correlation between PTBP1 expression in various cancer types and disease-specific survival (DSS) prognosis. Our analysis revealed that PTBP1 expression is associated with prognosis in nine types of cancer, and its expression can reasonably predict 1-year, 3-year, and 5-year survival rates in seven of these cancer types. We also investigated the potential of PTBP1 as an independent prognostic marker for glioma. To this end, we conducted univariate and multifactorial Cox regression analyses in four datasets, including TCGA-LGG/GBM, CGGA mRNA_301, CGGA mRNA_325, and CGGA mRNA_693. Although our results showed that PTBP1 expression is not an independent risk factor in the TCGA-LGG/GBM and CGGA mRNA_301 datasets, it was identified as an independent risk factor in the CGGA mRNA_325 and CGGA mRNA_693 datasets. However, given the relatively low hazard ratio (between 1.0 and 1.01) in the latter two datasets, we do not consider PTBP1 a reliable independent prognostic factor for glioma. This is primarily due to the strong correlation between PTBP1 expression and WHO grade and IDH1 mutation, both established high-risk factors across the four independent cohorts.

Next, we explored the impact of PTBP1 expression in low-grade gliomas. We first performed GSEA on differential expression genes in the PTBP1 high and low expression groups and found significant activation of cell cycle, cancer-related signalling pathways in the PTBP1 high expression group. Secondly, we found that PTBP1 expression was significantly lower in LGG patients with IDH1 mutant phenotype and that IDH1 wild type was also predominantly found in LGG patients with high PTBP1 expression. In the PTBP1 high expression group, the mutation rate of IDH1, MUC16, EGFR, NF1, PTEN, FLG and LRP2 genes were significantly higher. An increase in the number of mutated genes indicates an increase in the degree of LGG progression and represents an increased risk of death in LGG patients^65^. These findings highlight a correlation between mutations in IDH1 and PTBP1 expression. Consistent results were obtained across three independent CGGA glioma cohorts, reinforcing PTBP1 expression as a potential molecular marker for assessing LGG progression and prognosis.

Furthermore, we explored the link between methylation of PTBP1 and gene expression in LGG patients and clinical data. We found that methylation of PTBP1 decreased PTBP1 expression, although the mean methylation of PTBP1 did not correlate significantly with its expression. Based on this result, we speculate that the key methylation site that regulates PTBP1 expression may not be in the exons of PTBP1. Using the MEXPRESS database, we identified that methylation of the cg24840300 site downstream of PTBP1 was highly positively correlated with PTBP1 expression. This finding indicates a potential regulatory role of this methylation site in the gene expression pathways involving PTBP1, warranting further investigation.

We have demonstrated using immunohistochemistry in gliomas of different grades that PTBP1 protein expression rises with glioma progression. Using single-cell sequencing data, it was demonstrated that PTBP1 expression was not significantly cell-specific (Fig. S10), but was highest in AC-like malignant. AVIL is a newly identified glioma driver gene^66^. We calculated the correlation between AVIL expression and PTBP1 expression in GBM and LGG using the TIMER2.0 database. The results demonstrated that AVIL showed significant correlation with PTBP1 in both GBM and LGG (Fig. S11). This result further demonstrates the relationship between PTBP1 and glioma development and progression.

In summary, PTBP1 emerges as a promising prognostic marker across diverse cancer types. Particularly in low-grade glioma (LGG), PTBP1 shows significant positive correlations with critical tumor characteristics such as neoantigen load, tumor mutation burden, and immune cell infiltration. These correlations suggest that PTBP1 could serve as an indicator of disease progression and patient prognosis in LGG. Furthermore, the relationship between PTBP1 methylation levels and its expression levels has been found to be robust, with higher degrees of PTBP1 methylation correlating with improved patient survival outcomes. This observation underscores the prognostic significance of PTBP1 and its potential utility as a predictive molecular marker for glioma progression and survival. Our study elucidates the complex roles of PTBP1 in cancer biology, highlighting its value as a biomarker for a variety of cancer types. The positive associations observed between PTBP1 and tumor immune features within LGG cohorts indicate its potential role in modulating the tumor microenvironment. Additionally, our findings highlight the prognostic importance of PTBP1 methylation, which may offer a promising target for the development of future therapeutic interventions aimed at this oncogene.

Ultimately, the comprehensive characterization of PTBP1 in various cancer types paves the way for precision medicine approaches and personalized treatment strategies. Further elucidation of the underlying mechanisms governing PTBP1's role in cancer development and progression holds the potential to revolutionize cancer diagnostics and therapeutic interventions in the future.

### Supplementary Information

Supplementary Information 1.

Supplementary Information 2.

## Data Availability

All data generated and described in this article are available from the corresponding web servers, and are freely available to any scientist wishing to use them for noncommercial purposes, without breaching participant confidentiality. Further information is available from the corresponding author on reasonable request. All data from TCGA is available at their website: https://tcga-data.nci.nih.gov/. All data from CGGA is available at their website: http://cgga.org.cn/.

## Abbreviations

DSS: Disease-specific survival
BP: Biological process
OS: Overall survival
GSEA: Gene set enrichment analysis
SMART: Shiny methylation analysis resource tool
GTEx: Genotype-Tissue Expression Project
TCGA: The Cancer Genome Atlas
ACC: Adrenocortical carcinoma
BLCA: Bladder urothelial carcinoma
BRCA: Breast invasive carcinoma
CESC: Cervical squamous cell carcinoma and endocervical adenocarcinoma
CHOL: Cholangiocarcinoma
COAD: Colon adenocarcinoma
DLBC: Lymphoid neoplasm diffuse large B-cell lymphoma
ESCA: Esophageal carcinoma
GBM: Glioblastoma multiforme
GBMLGG: Glioma
HNSC: Head and neck squamous cell carcinoma
KICH: Kidney chromophobe
KIRC: Kidney renal clear cell carcinoma
KIRP: Kidney renal papillary cell carcinoma
LAML: Acute myeloid leukemia
LGG: Brain lower grade glioma
LIHC: Liver hepatocellular carcinoma
LUAD: Lung adenocarcinoma
LUSC: Lung squamous cell carcinoma
MESO: Mesothelioma
OV: Ovarian serous cystadenocarcinoma
PAAD: Pancreatic adenocarcinoma
PCPG: Pheochromocytoma and paraganglioma
PRAD: Prostate adenocarcinoma
READ: Rectum adenocarcinoma
STAD: Stomach adenocarcinoma
SKCM: Skin cutaneous melanoma
STES: Stomach and esophageal carcinoma
TGCT: Testicular germ cell tumors
THCA: Thyroid carcinoma
THYM: Thymoma
UCEC: Uterine corpus endometrial carcinoma
UVM: Uveal melanoma

## References

[1] Takahashi H, Nishimura J, Kagawa Y, Kano Y, Takahashi Y, Wu X, Hiraki M, Hamabe A, Konno M, Haraguchi N (2015). Significance of polypyrimidine tract-binding protein 1 expression in colorectal cancer. Mol. Cancer Ther..

[2] Sveen A, Kilpinen S, Ruusulehto A, Lothe RA, Skotheim RI (2016). Aberrant RNA splicing in cancer; expression changes and driver mutations of splicing factor genes. Oncogene.

[3] Han W, Wang L, Yin B, Peng X (2014). Characterization of a novel posttranslational modification in polypyrimidine tract-binding proteins by SUMO1. BMB Rep..

[4] Shinohara H, Kumazaki M, Minami Y, Ito Y, Sugito N, Kuranaga Y, Taniguchi K, Yamada N, Otsuki Y, Naoe T (2016). Perturbation of energy metabolism by fatty-acid derivative AIC-47 and imatinib in BCR-ABL-harboring leukemic cells. Cancer Lett..

[5] Zhu W, Zhou BL, Rong LJ, Ye L, Xu HJ, Zhou Y, Yan XJ, Liu WD, Zhu B, Wang L (2020). Roles of PTBP1 in alternative splicing, glycolysis, and oncogensis. J. Zhejiang Univ. Sci. B.

[6] Li J, Wang Y, Meng X, Liang H (2018). Modulation of transcriptional activity in brain lower grade glioma by alternative splicing. PeerJ.

[7] Hatoum A, Mohammed R, Zakieh O (2019). The unique invasiveness of glioblastoma and possible drug targets on extracellular matrix. Cancer Manag. Res..

[8] Majores M, von Lehe M, Fassunke J, Schramm J, Becker AJ, Simon M (2008). Tumor recurrence and malignant progression of gangliogliomas. Cancer.

[9] Dunn GP, Cloughesy TF, Maus MV, Prins RM, Reardon DA, Sonabend AM (2020). Emerging immunotherapies for malignant glioma: From immunogenomics to cell therapy. Neuro Oncol..

[10] Boussiotis VA, Charest A (2018). Immunotherapies for malignant glioma. Oncogene.

[11] Maxwell R, Luksik AS, Garzon-Muvdi T, Lim M (2017). The Potential of cellular- and viral-based immunotherapies for malignant glioma-dendritic cell vaccines, adoptive cell transfer, and oncolytic viruses. Curr. Neurol. Neurosci. Rep..

[12] Duan W-C, Wang L, Li K, Wang W-W, Zhan Y-B, Zhang F-J, Yu B, Bai Y-H, Wang Y-M, Ji Y-C (2018). IDH mutations but not TERTp mutations are associated with seizures in lower-grade gliomas. Medicine.

[13] Gillet E, Alentorn A, Doukouré B (2014). TP53 and p53 statuses and their clinical impact in diffuse low grade gliomas. J. Neuro-oncol..

[14] Wang YY, Zhang T, Li SW, Qian TY, Jiang T (2015). Mapping p53 mutations in low-grade glioma: A voxel-based neuroimaging analysis. Ajnr Am. J. Neuroradiol..

[15] Kannan K, Inagaki A, Silber J, Gorovets D, Huse JT (2012). Whole-exome sequencing identifies ATRX mutation as a key molecular determinant in lower-grade glioma. Oncotarget.

[16] Goldman M, Craft B, Kamath A, Brooks AN, Zhu J, Haussler D (2018). The UCSC Xena Platform for cancer genomics data visualization and interpretation. bioRxiv.

[17] Weinstein JN, Collisson EA, Mills GB, Shaw KRM, Ozenberger B, Ellrott K, Sander C, Stuart JM, Chang K, Creighton CJ (2013). The cancer genome atlas pan-cancer analysis project. Nat. Genet..

[18] Lonsdale J, Thomas J, Salvatore M, Phillips R, Lo E, Shad S, Hasz R, Walters G, Garcia F, Young N (2013). The Genotype-Tissue Expression (GTEx) project. Nat. Genet..

[19] Li Y, Ge D, Lu C (2019). The SMART App: An interactive web application for comprehensive DNA methylation analysis and visualization. Epigenet. Chromatin.

[20] Thorsson V, Gibbs DL, Brown SD, Wolf DM, Bortone DS, Yang TO, Portapardo E, Gao GF, Plaisier CL, Eddy JA (2018). The immune landscape of cancer. Immunity.

[21] Yang W, Soares J, Greninger P, Edelman EJ, Lightfoot H, Forbes S, Bindal N, Beare D, Smith JA, Thompson IR (2013). Genomics of Drug Sensitivity in Cancer (GDSC): A resource for therapeutic biomarker discovery in cancer cells. Nucleic Acids Res..

[22] Cheah, J. H., Bridger, H. S., Shamji, A. F., Schreiber, S. L. & Clemons, P. A. *Cancer Therapeutics Response Portal: A CTD*^*2*^* Network Resource for Mining Candidate Cancer Dependencies*.

[23] Becht E, Giraldo NA, Lacroix L, Buttard B, Elarouci N, Petitprez F, Selves J, Laurentpuig P, Sautesfridman C, Fridman WH (2016). Estimating the population abundance of tissue-infiltrating immune and stromal cell populations using gene expression. Genome Biol..

[24] Yoshihara K, Shahmoradgoli M, Martinez E, Vegesna R, Kim H, Torresgarcia W, Trevino V, Shen H, Laird PW, Levine DA (2013). Inferring tumour purity and stromal and immune cell admixture from expression data. Nat. Commun..

[25] Li T, Fan J, Wang B, Traugh N, Chen Q, Liu JS, Li B, Liu XS (2017). TIMER: A web server for comprehensive analysis of tumor-infiltrating immune cells. Cancer Res..

[26] Newman AM (2019). Determining cell type abundance and expression from bulk tissues with digital cytometry. Nat. Biotechnol..

[27] Subramanian A, Tamayo P, Mootha VK, Mukherjee S, Ebert BL, Gillette MA, Paulovich A, Pomeroy SL, Golub TR, Lander ES (2005). Gene set enrichment analysis: A knowledge-based approach for interpreting genome-wide expression profiles. Proc. Natl. Acad. Sci. USA.

[28] Aleksander SA, Balhoff J, Carbon S, Cherry JM, Drabkin HJ, Ebert D, Feuermann M, Gaudet P, Harris NL (2023). The gene ontology knowledgebase in 2023. Genetics.

[29] Hanahan D (2022). Hallmarks of cancer: New dimensions. Cancer Discov..

[30] Liberzon A, Birger C, Thorvaldsdóttir H, Ghandi M, Mesirov JP, Tamayo P (2015). The molecular signatures database hallmark gene set collection. Cell Syst..

[31] Vasaikar SV, Straub P, Wang J, Zhang B (2018). LinkedOmics: Analyzing multi-omics data within and across 32 cancer types. Nucleic Acid Res..

[32] Filbin MG, Tirosh I, Hovestadt V, Shaw ML, Escalante LE, Mathewson ND, Neftel C, Frank N, Pelton K, Hebert CM (2018). Developmental and oncogenic programs in H3K27M gliomas dissected by single-cell RNA-seq. Science.

[33] Butler A, Hoffman P, Smibert P, Papalexi E, Satija R (2018). Integrating single-cell transcriptomic data across different conditions, technologies, and species. Nat. Biotechnol..

[34] Kassambara, A., Kosinski, M. & Biecek, P. *survminer: Drawing Survival Curves using 'ggplot2'* (2016).

[35] Robin X, Turck N, Hainard A, Tiberti N, Lisacek F, Sanchez JC, Müller M (2011). pROC: an open-source package for R and S+ to analyze and compare ROC curves. Bmc Bioinform..

[36] Li GM (2008). Mechanisms and functions of DNA mismatch repair. Cell Res..

[37] Moore LD, Le T, Fan G (2013). DNA methylation and its basic function. Neuropsychopharmacology.

[38] Fuks F, Burgers WA, Brehm A, Hughes-Davies L, Kouzarides T (2000). DNA methyltransferase Dnmt1 associates with histone deacetylase activity. Nat. Genet..

[39] Lyko F (2018). The DNA methyltransferase family: A versatile toolkit for epigenetic regulation. Nat. Rev. Genet..

[40] Ma S, Chee J, Fear VS, Forbes CA, Boon L, Dick IM, Robinson BWS, Creaney J (2020). Pre-treatment tumor neo-antigen responses in draining lymph nodes are infrequent but predict checkpoint blockade therapy outcome. Oncoimmunology.

[41] Esprit A, de Mey W, Bahadur Shahi R, Thielemans K, Franceschini L, Breckpot K (2020). Neo-antigen mRNA vaccines. Vaccines (Basel).

[42] Galuppini F, Dal Pozzo CA, Deckert J, Loupakis F, Fassan M, Baffa R (2019). Tumor mutation burden: From comprehensive mutational screening to the clinic. Cancer Cell Int..

[43] Wu Y, Xu J, Du C, Wu Y, Xia D, Lv W, Hu J (2019). The Predictive value of tumor mutation burden on efficacy of immune checkpoint inhibitors in cancers: A systematic review and meta-analysis. Front. Oncol..

[44] Offin M, Rizvi H, Tenet M, Ni A, Sanchez-Vega F, Li BT, Drilon A, Kris MG, Rudin CM, Schultz N (2019). Tumor mutation burden and efficacy of EGFR-tyrosine kinase inhibitors in patients with EGFR-mutant lung cancers. Clin. Cancer Res..

[45] Sharma P, Allison JP (2015). The future of immune checkpoint therapy. Science.

[46] Charoentong P, Finotello F, Angelova M, Mayer C, Efremova M, Rieder D, Hackl H, Trajanoski Z (2017). Pan-cancer immunogenomic analyses reveal genotype-immunophenotype relationships and predictors of response to checkpoint blockade. Cell Rep..

[47] Stanton SE, Disis ML (2016). Clinical significance of tumor-infiltrating lymphocytes in breast cancer. J. Immunother. Cancer.

[48] Cheng W, Ren X, Zhang C, Cai J, Han S, Wu A (2017). Gene Expression profiling stratifies IDH1-mutant glioma with distinct prognoses. Mol. Neurobiol..

[49] Taniguchi K, Sugito N, Shinohara H, Kuranaga Y, Inomata Y, Komura K, Uchiyama K, Akao Y (2018). Organ-specific microRNAs (MIR122, 137, and 206) contribute to tissue characteristics and carcinogenesis by regulating pyruvate kinase M1/2 (PKM) expression. Int. J. Mol. Sci..

[50] Ramos AD, Andersen RE, Liu SJ, Nowakowski TJ, Hong SJ, Gertz C, Salinas RD, Zarabi H, Kriegstein AR, Lim DA (2015). The long noncoding RNA Pnky regulates neuronal differentiation of embryonic and postnatal neural stem cells. Cell Stem Cell.

[51] Baretti M, Le DT (2018). DNA mismatch repair in cancer. Pharmacol. Ther..

[52] Ijsselsteijn R, Jansen JG, de Wind N (2020). DNA mismatch repair-dependent DNA damage responses and cancer. DNA Repair..

[53] Sharma P, Siddiqui BA, Anandhan S, Yadav SS, Subudhi SK, Gao J, Goswami S, Allison JP (2021). The next decade of immune checkpoint therapy. Cancer Discov..

[54] Snyder A, Makarov V, Merghoub T, Yuan J, Zaretsky JM, Desrichard A, Walsh LA, Postow MA, Wong P, Ho TS (2014). Genetic basis for clinical response to CTLA-4 blockade in melanoma. N. Engl. J. Med..

[55] Rizvi NA, Hellmann MD, Snyder A, Kvistborg P, Makarov V, Havel JJ, Lee W, Yuan J, Wong P, Ho TS (2015). Mutational landscape determines sensitivity to PD-1 blockade in non–small cell lung cancer. Science.

[56] Hugo W, Zaretsky JM, Sun L, Song C, Moreno BH, Hu-Lieskovan S, Berent-Maoz B, Pang J, Chmielowski B, Cherry G (2016). Genomic and transcriptomic features of response to anti-PD-1 therapy in metastatic melanoma. Cell.

[57] Carbone DP (2017). First-line nivolumab in stage IV or recurrent non–small-cell lung cancer. N. Engl. J. Med..

[58] Matsushita H, Vesely MD, Koboldt DC, Rickert CG, Uppaluri R, Magrini VJ, Arthur CD, White JM, Chen YS, Shea LK (2012). Cancer exome analysis reveals a T-cell-dependent mechanism of cancer immunoediting. Nature.

[59] Riaz N, Morris L, Havel JJ, Makarov V, Desrichard A, Chan TA (2016). The role of neoantigens in response to immune checkpoint blockade. Int. Immunol..

[60] Ott PA, Hu Z, Keskin DB, Shukla SA, Sun J, Bozym DJ, Zhang W, Luoma A, Giobbie-Hurder A, Peter L (2017). An immunogenic personal neoantigen vaccine for patients with melanoma. Nature.

[61] Deng L, Gyorffy B, Na F, Chen B, Lan J, Xue J, Zhou L, Lu Y (2015). Association of PDCD1 and CTLA-4 gene expression with clinicopathological factors and survival in non–small-cell lung cancer: Results from a large and pooled microarray database. J. Thorac. Oncol..

[62] Guo J, Xue Z, Wang L (2022). Transcriptional regulation of the immune checkpoints PD-1 and CTLA-4. Cell. Mol. Immunol..

[63] Zhang H, Dai Z, Wu W, Wang Z, Zhang N, Zhang L, Zeng W-J, Liu Z, Cheng Q (2021). Regulatory mechanisms of immune checkpoints PD-L1 and CTLA-4 in cancer. J. Exp. Clin. Cancer Res..

[64] Li L, Zhang M, Zhu D, Wang X (2021). High expression of cluster of differentiation 276 indicates poor prognosis in glioma. Clin. Med. Insights Oncol..

[65] Cancer Genome Atlas Research Network (2015). Comprehensive, integrative genomic analysis of diffuse lower-grade gliomas. N. Engl. J. Med..

[66] Xie Z, Janczyk PL, Zhang Y, Liu A, Shi X, Singh S, Facemire L, Kubow K, Li Z, Jia Y (2020). A cytoskeleton regulator AVIL drives tumorigenesis in glioblastoma. Nat. Commun..

