# Supplementary material for: Pan-cancer multi-omics analysis of PTBP1 reveals it as an inflammatory, progressive and prognostic marker in glioma
Source: Sci Rep. 2024 Jun 25;14:14584. doi: 10.1038/s41598-024-64979-5 (PMC11199703; doi:10.1038/s41598-024-64979-5)
Supplement: Supplementary file 1 — Supplementary Information 1. [file 41598_2024_64979_MOESM1_ESM.pdf]

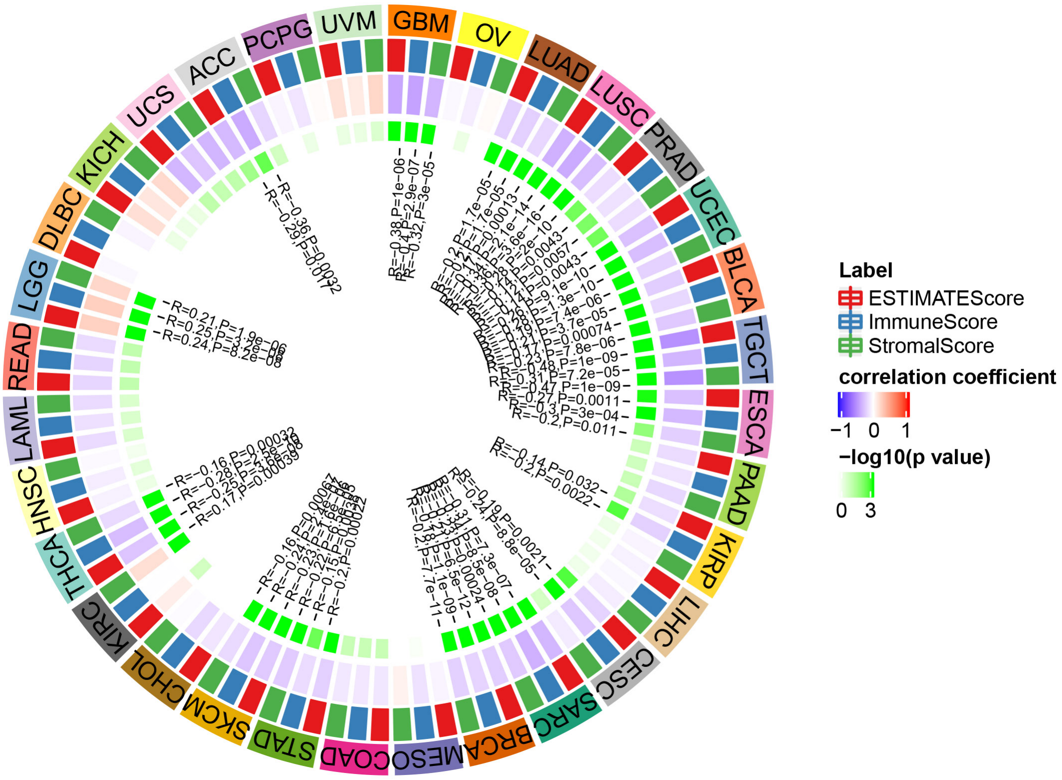

Figure S1. The Pearson correlation between the immune scores of 33 cancers in the TCGA database obtained by the ESTIMATE algorithm and the PTBP1 expression.

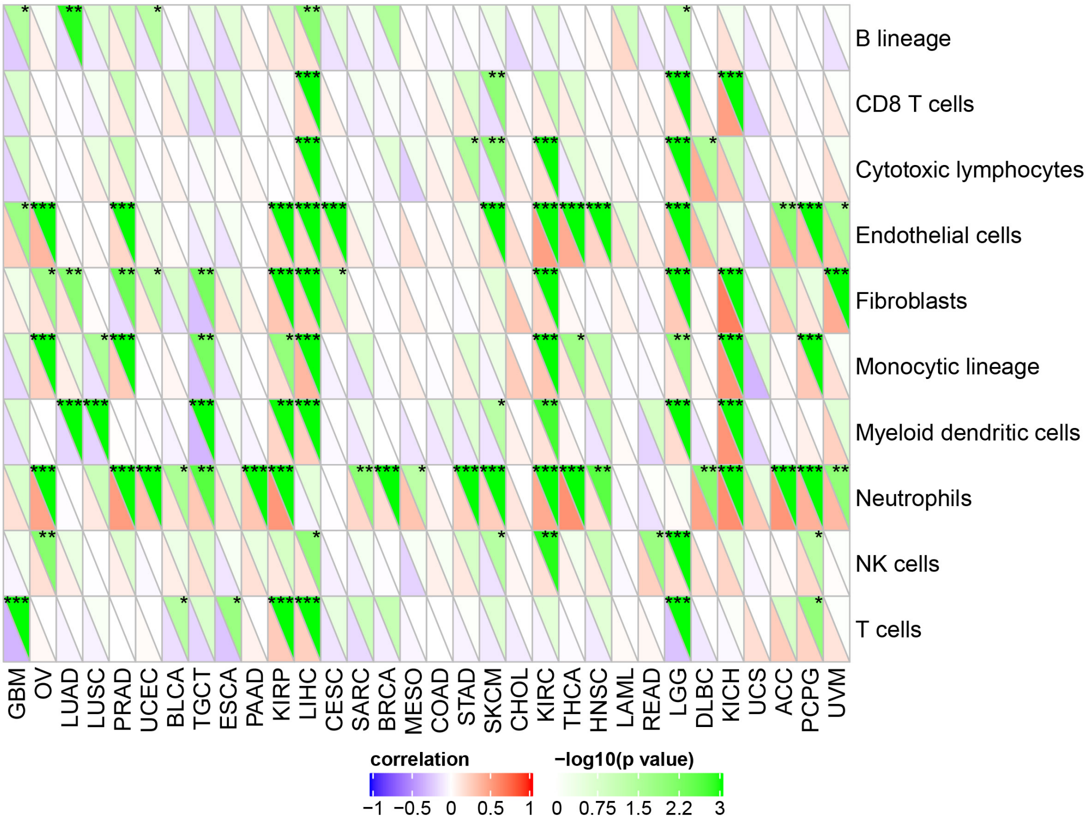

Figure S2. The Pearson correlation between the immune scores of 33 cancers in the

TCGA database obtained by the MCPcounter algorithm and the PTBP1 expression.

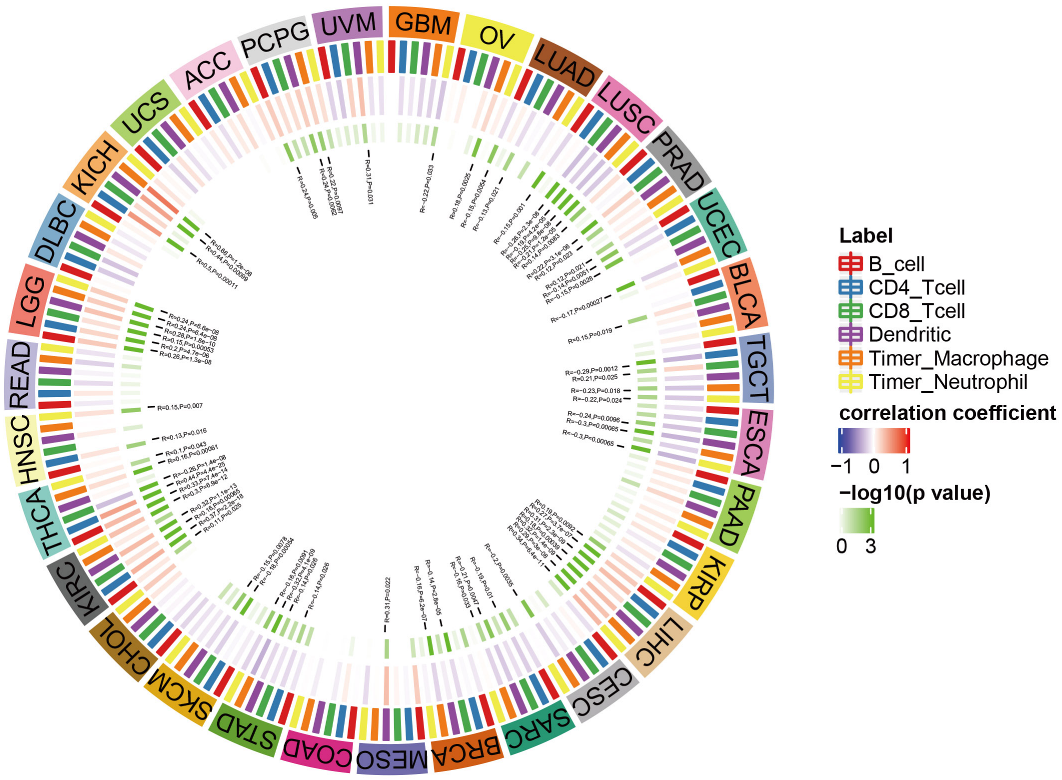

Figure S3. The Pearson correlation between the immune scores of 33 cancers in the TCGA database obtained by the TIMER database and the PTBP1 expression.

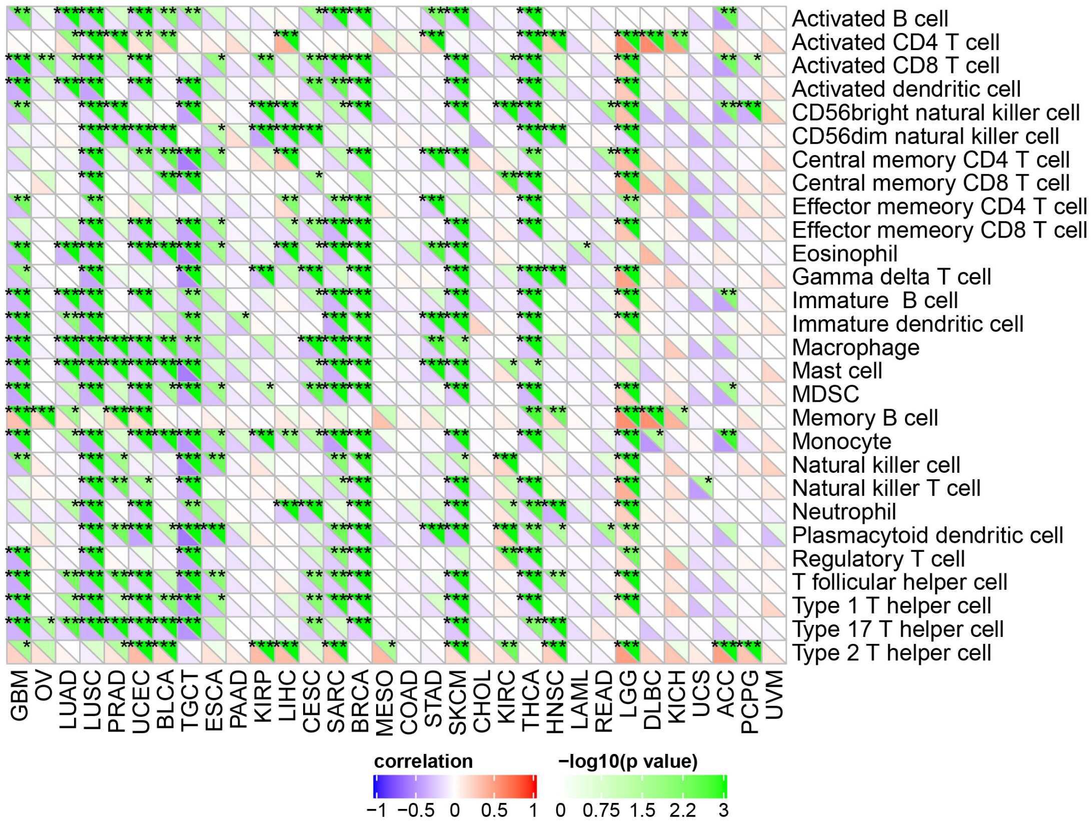

Figure S4. The Pearson correlation between the immune scores of 33 cancers in the TCGA database obtained by the CIBERSORT algorithm and the the PTBP1 expression.

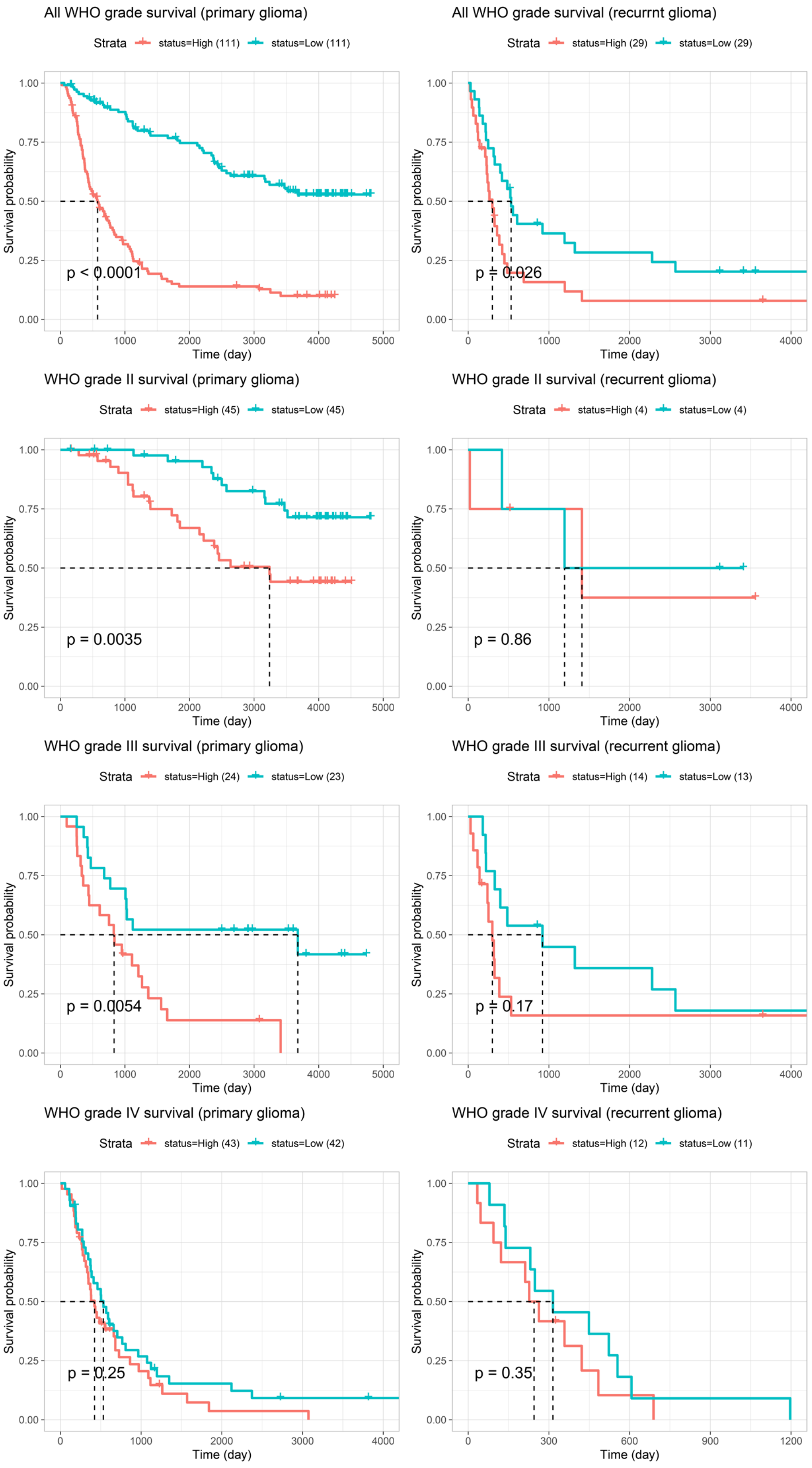

Figure S5. Relationship between PTBP1 expression and prognosis for the mRNA_325 dataset in the CGGA database.

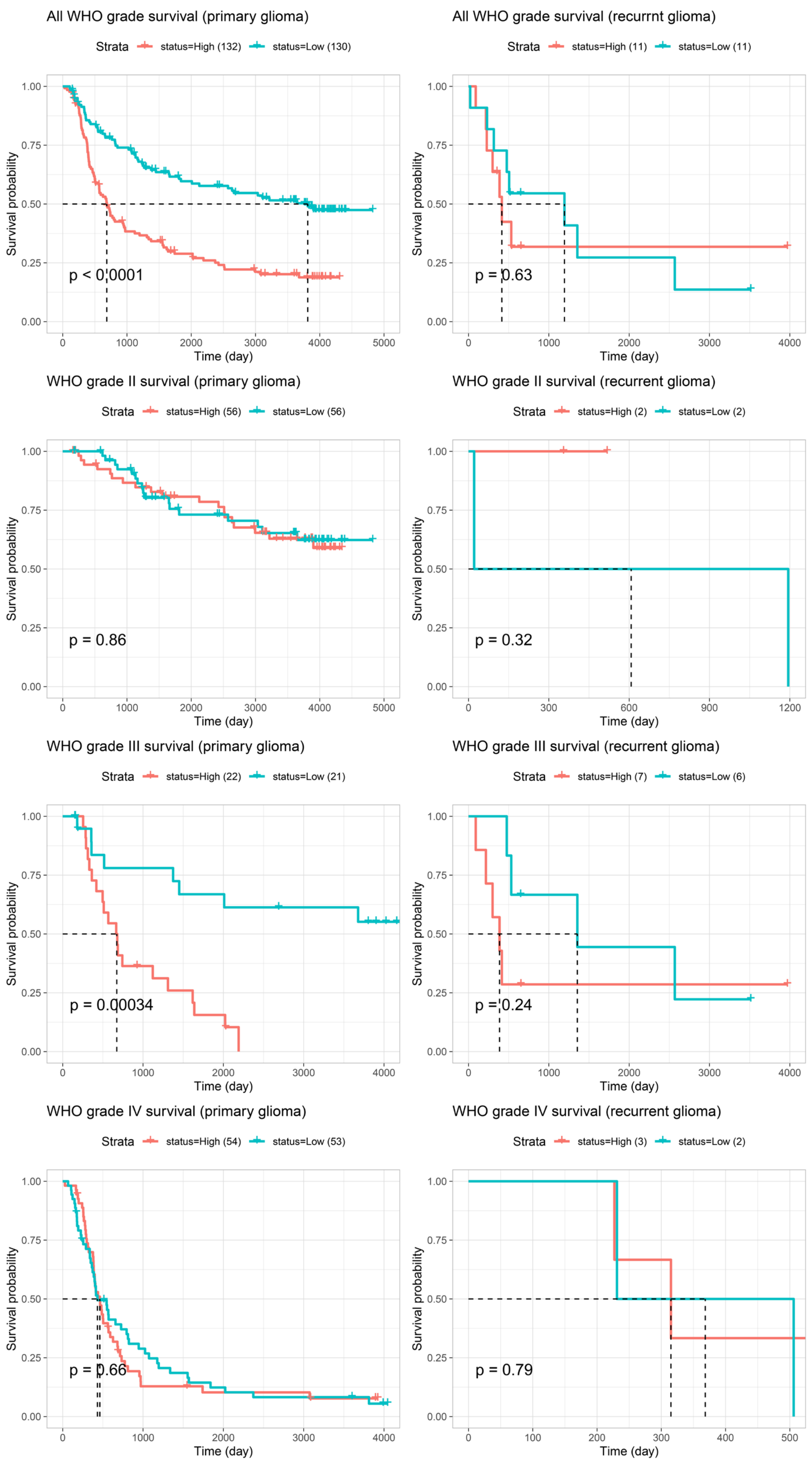

Figure S6. Relationship between PTBP1 expression and prognosis for the mRNA_301 dataset.

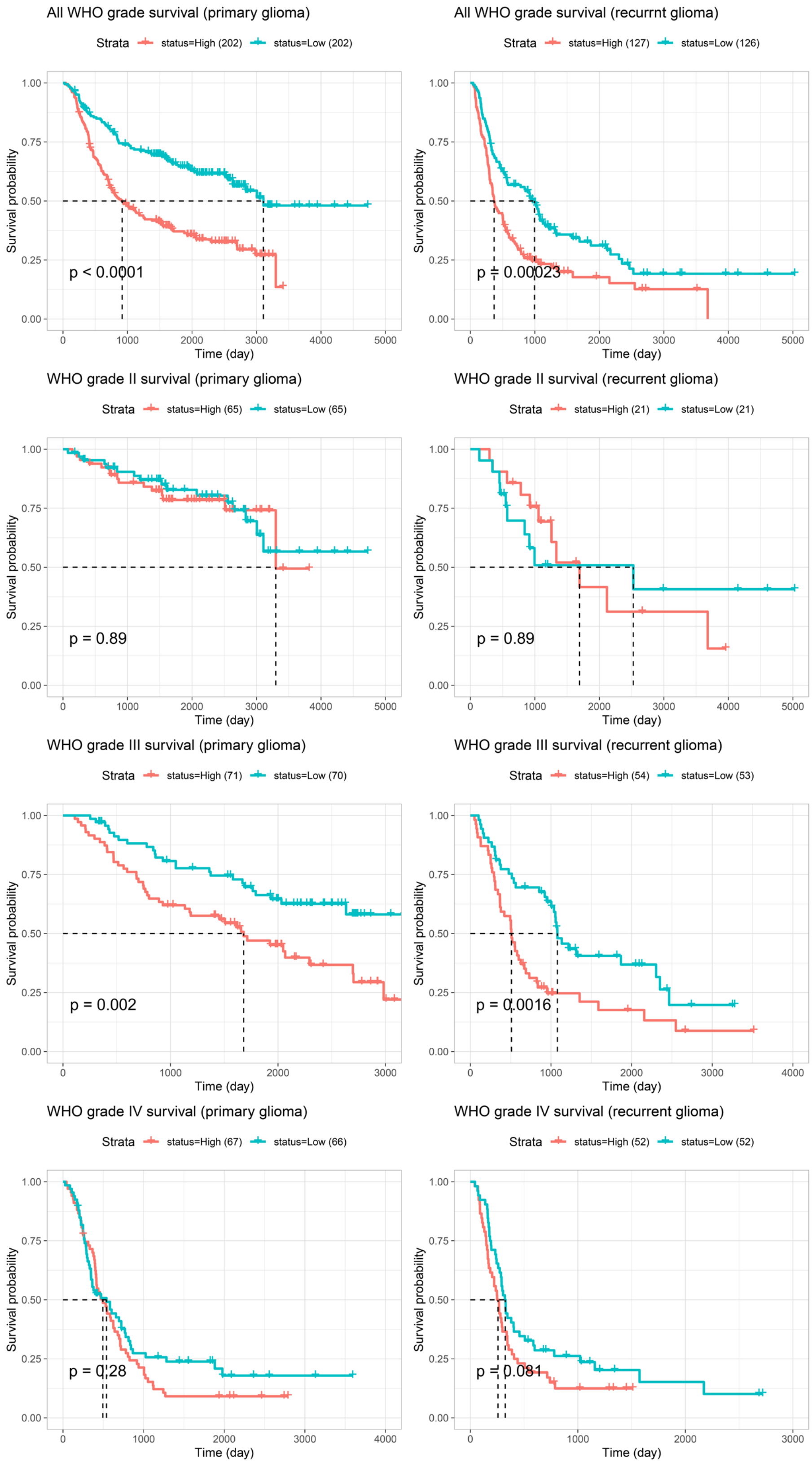

Figure S7. Relationship between PTBP1 expression and prognosis in the mRNA_693 dataset in the CGGA database.

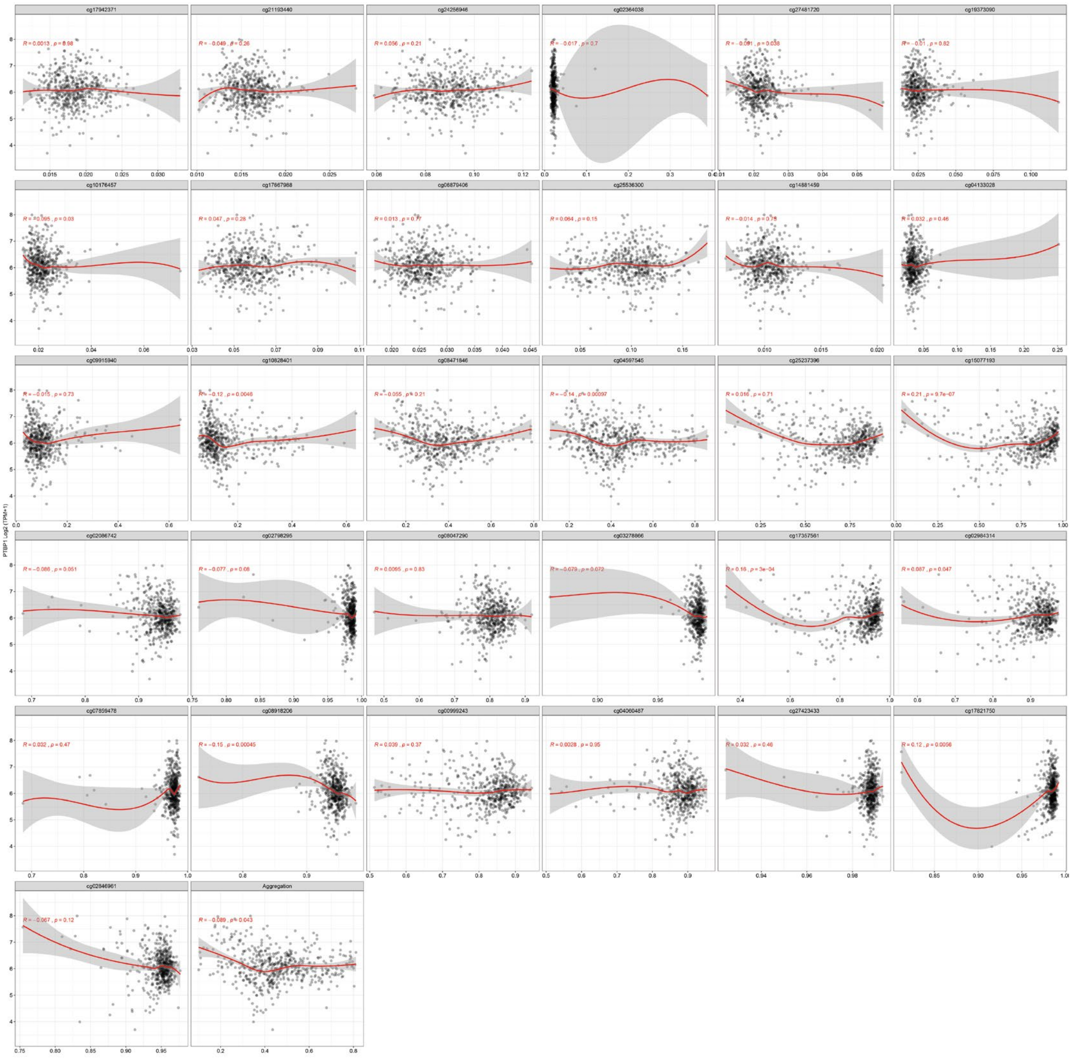

Figure S8. Analysis of the correlation between the PTBP1 expression and the methylation of its 31 methylation sites.

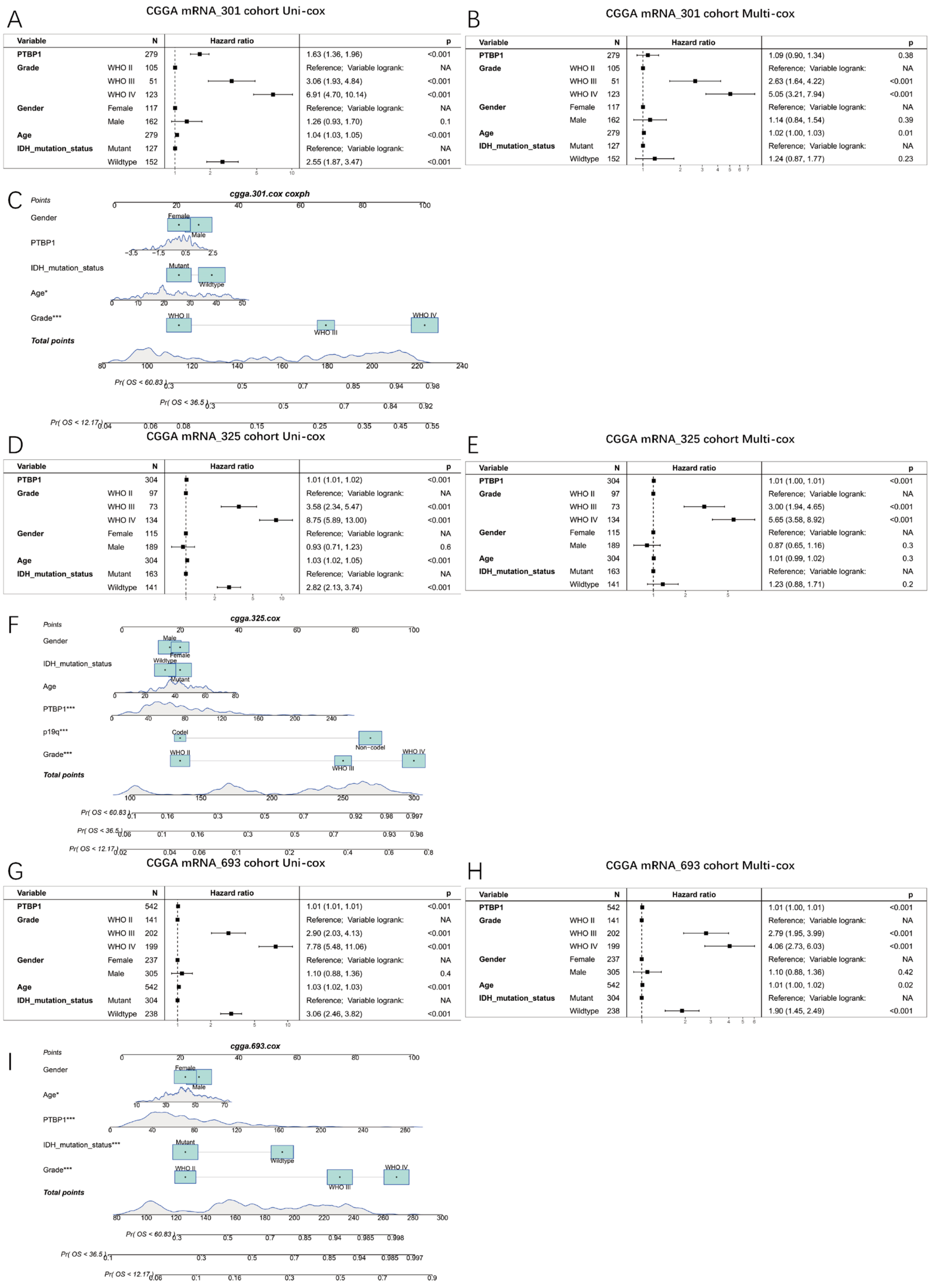

Figure S9. Validation of PTBP1 as an independent prognostic factor for glioma in three independent cohorts of CGGA mRNA_301, CGGA mRNA_325, CGGA mRNA_693. (A-C) unicox, multicox forest plot and nomogram of CGGA mRNA_301 cohort. (D-F) Unicox, multicox forest plot and nomogram of CGGA mRNA_325 cohort. (G-I) CGGA mRNA_693 cohort of unicox, multicox forest plots and nomogram.

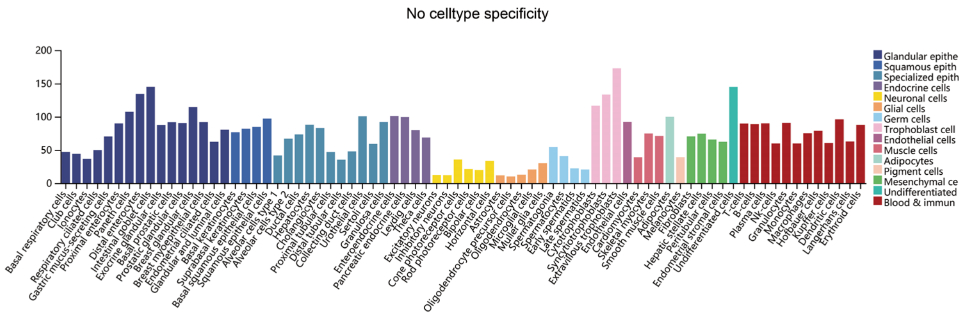

Figure S10. The expression of PTBP1 in a variety of cell types was obtained on the The human protein atlas website. The results indicate that PTBP1 is not cell type specific.

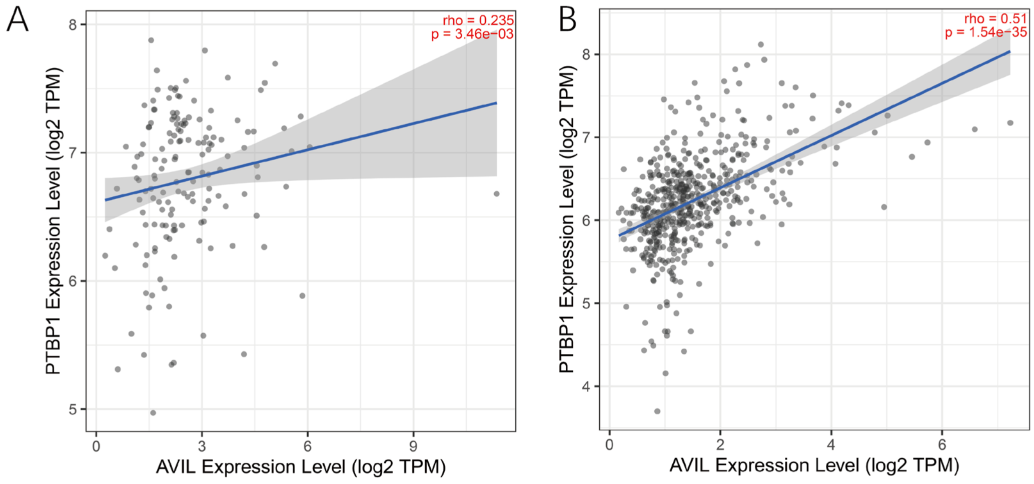

Figure S11. Correlation of AVIL with PTBP1 expression.

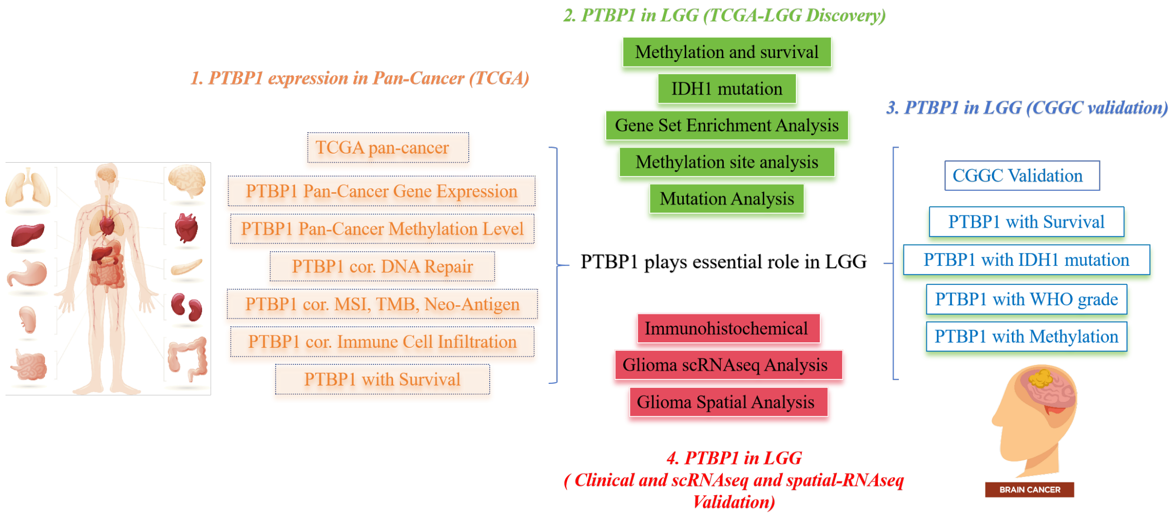

Figure S12. The workflow of the research.
